# SYBA: Bayesian estimation of synthetic accessibility of organic compounds

**DOI:** 10.1186/s13321-020-00439-2

**Published:** 2020-05-20

**Authors:** Milan Voršilák, Michal Kolář, Ivan Čmelo, Daniel Svozil

**Affiliations:** 1 CZ-OPENSCREEN: National Infrastructure for Chemical Biology, Department of Informatics and Chemistry, Faculty of Chemical Technology, University of Chemistry and Technology Prague, Technická 5, 166 28 Prague 6, Czech Republic; 2 CZ-OPENSCREEN: National Infrastructure for Chemical Biology, Institute of Molecular Genetics of theCzech Academy of Sciences, Vídeňská 1083, 142 20 Prague 4, Czech Republic; 3 Laboratory of Genomics and Bioinformatics, Institute of Molecular Genetics, Czech of the Academy of Sciences, Vídeňská 1083, 142 20 Prague 4, Czech Republic; 4 Department of Informatics and Chemistry, Faculty of Chemical Technology, University of Chemistry and Technology Prague, Technická 5, 166 28 Prague 6, Czech Republic

**Keywords:** Synthetic accessibility, Bayesian analysis, Bernoulli naïve Bayes

## Abstract

SYBA (SYnthetic Bayesian Accessibility) is a fragment-based method for the rapid classification of organic compounds as easy- (ES) or hard-to-synthesize (HS). It is based on a Bernoulli naïve Bayes classifier that is used to assign SYBA score contributions to individual fragments based on their frequencies in the database of ES and HS molecules. SYBA was trained on ES molecules available in the ZINC15 database and on HS molecules generated by the Nonpher methodology. SYBA was compared with a random forest, that was utilized as a baseline method, as well as with other two methods for synthetic accessibility assessment: SAScore and SCScore. When used with their suggested thresholds, SYBA improves over random forest classification, albeit marginally, and outperforms SAScore and SCScore. However, upon the optimization of SAScore threshold (that changes from 6.0 to – 4.5), SAScore yields similar results as SYBA. Because SYBA is based merely on fragment contributions, it can be used for the analysis of the contribution of individual molecular parts to compound synthetic accessibility. SYBA is publicly available at https://github.com/lich-uct/syba under the GNU General Public License.

## Background

Chemical space available for the generation of new molecules is huge [1–4], making the synthesis and testing of all possible compounds impractical. Therefore chemists, both experimental and computational, developed tools and approaches for the exploration of chemical space with the aim to identify new compounds with desirable physico-chemical, biological and pharmacological properties [5–12]. A major in silico method for chemical space exploration is de novo molecular design in which new virtual molecules are assembled from scratch [13–18]. An essential requirement for de novo designed compounds is their synthetic accessibility. Synthetic accessibility is commonly incorporated into de novo design programs by employing chemical strategies that guide an assembly process. For example, the connections between certain atom types can be disallowed [19], established chemical reactions can be used to connect individual molecular building blocks [20, 21] or the retrosynthetic rules can be directly incorporated into the assembly process [22, 23].

The latest development in de novo molecular design are molecular generators based on deep learning approaches [24–26]. These typically construct new molecules not by assembling the building blocks, but by producing chemically feasible SMILES strings [27–32]. The generators are able to produce millions of virtual compounds, synthetic accessibility of which has to be quickly and efficiently assessed. Quick synthetic accessibility assessment can be based [33] on molecule’s complexity that is typically calculated [34–37] from the number of atoms, bonds, rings, and/or hard-to-synthesize motifs, such as chiral centers or uncommon ring fusions. However, the definition of molecular complexity is ambiguous and context dependent [38, 39]. The structural complexity is not equivalent to the synthetic one as complexity-based metrics do not incorporate any information about starting materials and tend to remove molecules that can be synthesized from already existing complex precursors [40, 41]. A better way of synthetic accessibility assessment is to use the complexity of the synthetic route [42]. Based on this principle, SCScore, a data-driven metric designed to describe real syntheses, was developed recently [43]. SCScore is based on the idea that reaction products are synthetically more complex than reactants. To quantify this, a deep feed-forward neural network, that assigns a synthetic complexity score between 1 and 5, was trained on 22 million reactant-product pairs from the Reaxys database [44]. Using the hinge loss objective function, that supports the separation between scores in each reactant–product pair, the model learns synthetic complexity score that correlates with the number of reaction steps, but does not rely on the availability of reaction database or organic chemist ranking.

SAScore [45], another popular and rapid method for synthetic accessibility assessment, is based on the analysis of ECFP4 [46] fragments obtained from one million compounds randomly selected from the PubChem database [47]. The main idea of SAScore is that when a molecular fragment occurs often in the PubChem database, it contributes to the synthetic accessibility of a molecule more than a less frequently occurring fragment. Each fragment is assigned a numerical score, frequent fragments have positive scores and less frequent fragments have negative scores. In addition to the fragment score, SAScore consists of a complexity penalty and symmetry bonus. These terms penalize nonstandard structural motives such as macrocycles, stereo centers, spiro and bridge atom, but reward the symmetry of a structure. SAScore acquires values between 1 (easy to make) and 10 (very difficult to make), where 6.0 is suggested by the authors [45] as a threshold to distinguish between easy- and hard-to-synthesize compounds. SAScore is a popular high-throughput measure and proved to be a very useful tool in many cheminformatics applications [27, 48–50].

In the present work, we further expand on main concepts of SAScore construction. We developed SYBA (SYnthetic Bayesian Accessibility), a rapid fragment-based score derived using Bayesian probabilistic modeling. Fragment contributions to SYBA are calculated not only from fragments present in synthetically accessible molecules, but also from fragments appearing in hard-to-synthesize molecules.

## Methods

### SYBA score derivation

SYBA is a Bernoulli naïve Bayes classifier based on the frequency of molecular fragments that are present in the database of easy-to-synthesize (ES) and hard-to-synthesize (HS) molecules and on the assumption of the independence of molecular fragments. Though such assumption is bold, it was shown to provide surprisingly good results in many cheminformatics studies [51–55].

Each compound is represented by a binary fingerprint $\varvec{F} = \left[ {f_{1} ,f_{2} , \ldots ,f_{M} } \right]$ of length *M* where $f_{i}$ indicates the presence ($f_{i} = 1$) or absence ($f_{i} = 0$) of the specific fragment *i* in the compound. SYBA uses this fingerprint to assign the molecule to a class $C \in \left\langle {{\text{ES}},{\text{HS}}} \right\rangle$. The calculation is based on the Bayes theorem

1 $$p\left( {C |\varvec{F}} \right) = \frac{{p\left( {\varvec{F}|C} \right) p\left( C \right)}}{{p\left( \varvec{F} \right)}},$$

where $p\left( {C |\varvec{F}} \right)$ is the posterior probability that a compound with a certain set of molecular fragments ***F*** belongs to the class $C$. The likelihood $p(\varvec{F}|C)$ is the conditional probability that a compound from the class $C$ contains a set of molecular fragments ***F***. The marginal probabilities $p\left( \varvec{F} \right)$ and $p\left( C \right)$ express our belief to observe a set of molecular fragments ***F*** and the molecule that belongs to the class $C$.

The SYBA score is defined as the logarithm of the ratio of the posterior probabilities that the molecule belongs to the ES and HS classes,

2 $${\text{SYBA}}\left( \varvec{F} \right) = \ln \left( {\frac{{p\left( {{\text{ES|}}\varvec{F}} \right)}}{{p\left( {{\text{HS|}}\varvec{F}} \right)}}} \right) .$$

Using Eq. 1, the SYBA score can be expressed as

3 $${\text{SYBA}}\left( \varvec{F} \right) = \ln \left( {\frac{{p\left( {\text{ES}} \right)}}{{p\left( {\text{HS}} \right)}}} \right) + \ln \left( {\frac{{p\left( {\varvec{F} | {\text{ES}}} \right)}}{{p\left( {\varvec{F} | {\text{HS}}} \right)}}} \right) .$$

In the data set SYBA was derived from (further referred to as the training data set S), ES and HS compounds are represented evenly, the priors $p\left( {\text{ES}} \right)$ and $p\left( {\text{HS}} \right)$ are thus equal and the term $\ln \left( {\frac{{p\left( {\text{ES}} \right)}}{{p\left( {\text{HS}} \right)}}} \right)$ becomes zero:

4 $${\text{SYBA}}\left( \varvec{F} \right) = \ln \left( {\frac{{p\left( {\varvec{F} | {\text{ES}}} \right)}}{{p\left( {\varvec{F} | {\text{HS}}} \right)}}} \right) .$$

Assuming the independence of molecular fragments, the conditional probability $p\left( {\varvec{F} |C} \right)$ factorizes to $p\left( {\varvec{F} |C} \right) = \mathop \prod \limits_{i = 1}^{M} p(f_{i} |C)$ and the SYBA score simplifies to

5 $${\text{SYBA}}\left( \varvec{F} \right) = \sum\nolimits_{i = 1}^{M} {s_{i} \left( {f_{i} } \right)}$$

where $s_{i} \left( {f_{i} } \right)$ is the score contribution from the fragment *i* (SYBA fragment score) given as

6 $$s_{i} \left( {f_{i} } \right) = \ln \left( {\frac{{p\left( {f_{i} | {\text{ES}}} \right)}}{{p\left( {f_{i} | {\text{HS}}} \right)}}} \right).$$

Considering that $p\left( {f_{i} | {\text{ES}}} \right) = 1 - p\left( {f_{i} | {\text{HS}}} \right)$, the fragment scores $s_{i} \left( {f_{i} } \right)$ in Eq. 6 represent logits and can be expressed using the fragment frequencies in the training data set S as

7 $$s_{i} \left( {f_{i} } \right) = { \ln }\frac{{N_{\text{HS}} + 2}}{{N_{\text{ES}} + 2}} + f_{i} {\text{ln}}\frac{{\left( {n_{{{\text{ES}},i}} + 1} \right)}}{{\left( {n_{{{\text{HS}},i}} + 1} \right)}} + (1 - f_{i} ) { \ln }\frac{{\left( {N_{\text{ES}} - n_{{{\text{ES}},i}} + 1} \right)}}{{\left( {N_{\text{HS}} - n_{{{\text{HS}},i}} + 1} \right)}},$$

where $N_{\text{HS}}$ is the number of HS and $N_{\text{ES}}$ the number of ES molecules in the training data set S, $n_{{{\text{HS}},i}}$ is the number of HS molecules in the training data set S that contain the fragment *i,* and $n_{{{\text{ES}},i}}$ is the number of ES molecules in the training data set S that contain the fragment *i.* See Additional file 2 for a detailed derivation. Positive $s_{i} \left( {f_{i} } \right)$ means that the presence/absence of the fragment *i* is more probable in ES than in HS class and vice versa. Positive SYBA means that the compound belongs more likely to the ES class, while negative SYBA means that the compound belongs more likely to the HS class. The higher the absolute value of SYBA, the more evidence for the class membership is present in the molecule.

### Training set construction

**Fig. 1:**
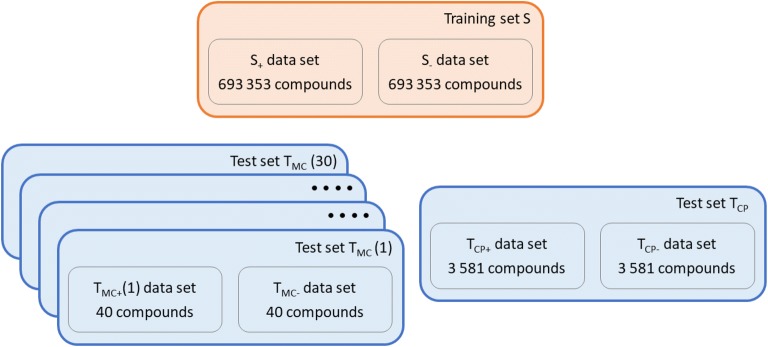
Data set summary. Training set was used to derive SYBA scores, as well as to train a random forest classifier. Training set consists of 693 353 molecules randomly selected from the ZINC15 database [57] that are considered to be ES (S_+_ data set) and of the same number of HS molecules generated by Nonpher [58] (S_−_ data set). Two test sets were used to compare the performance of SYBA, a random forest, SAScore [45] and SCScore [43]. Manually curated test set (T_MC_) contains 40 compounds (T_MC-_ data set) considered to be HS by experienced medicinal chemists [58] supplemented by 40 ES compounds randomly selected from the ZINC15 database (T_MC+_ data set). 30 T_MC_ data set instances differing in T_MC+_ compounds were constructed. Computationally picked test set (T_CP_) consists of 3 581 HS compounds that were obtained from the GDB-17 database [61] (T_CP-_ data set) complemented by the same number of compounds randomly selected from the ZINC15 database (T_CP+_ data set)

The training data set S consists of two subsets: S_+_ contains ES structures and S_-_ contains HS structures (Fig. 1, Additional file 1). While ES molecules can be readily obtained, for example, from the ZINC database of purchasable compounds [56, 57], no equivalent database of HS molecules exists. However, HS molecules can be designed by Nonpher [58], a method based on a molecular morphing approach [59]. In Nonpher, a starting molecule is gradually transformed into a more complex compound using small structural perturbations, such as the addition or removal of an atom or a bond. To prevent the creation of overly complex structures, four complexity indices (Bertz [34], Whitlock [35], BC [36] and SMCM [37]) are monitored and once their respective thresholds (Additional file 2: Table S1) are exceeded, Nonpher is stopped.

Using Nonpher, 693 353 HS molecules were generated and they form the S_-_ data set. The S_+_ data set, containing ES compounds, is formed by the same number of molecules randomly chosen (excluding natural products) from the ZINC15 database [57] so that their distribution of the number of heavy atoms is the same as in the S_-_ data sets. Every S_+_ and S_-_ molecule was fragmented using the Morgan fingerprint function in the RDKit toolkit [60]. Fragments with the radius of 4 and smaller, corresponding to radial ECFP8 [46] fragments, were used. This type of fragments consists of a central atom and atoms distant from the central atom up to four bonds. Besides ECFP8 fragments, the number of stereocenters was also included into SYBA as the molecules with more stereocenters are typically more difficult to synthesize. The stereo score is based, similarly to the fragment score, on the analysis of the number of stereocenters in the training set S. To obtain the stereo score, molecules were divided into 6 bins differing by the number of stereocenters (0, 1, 2, 3, 4 and 5+) and individual score contributions were calculated from Eq. 7.

### Test set construction

SYBA performance could have been assessed using a test set created in a similar way as the training set S, i.e. using HS compounds generated by Nonpher. However, such test set would be clearly biased towards chemical space covered by Nonpher. Therefore, two test sets were constructed in a conceptually different manner. First test set, further denoted as T_MC_, was manually curated from the literature, second test set, referred to as T_CP_, was computationally picked from the ZINC15 [57] and GDB17 databases [61].

HS compounds in T_MC_ (denoted as T_MC−_) were obtained by the analysis [58] of 296 published compounds assessed by experienced medicinal chemists [41, 45, 62, 63]. Based on original chemists’ scores, the final T_MC-_ data set of 40 HS compounds was assembled. A complementary T_MC+_ data set consists of 40 ES compounds selected from the ZINC15 database [57] in such a way that the distribution of the number of their heavy atoms is the same as in the T_MC-_ data set. Because small T_MC_ size may bias the results, 30 different T_MC_ data set instances were generated using the same 40 T_MC-_ compounds, but different 40 T_MC+_ compounds (Additional file 3).

HS compounds in the T_CP_ test set (Additional file 4), denoted as T_CP-_, were obtained by the analysis of the publicly available subset of 50 M molecules from the GDB-17 database [61]. Only molecules exceeding thresholds (Additional file 2: Table S1) of all monitored complexity indices (Bertz [34], Whitlock [35], BC [36] and SMCM [37]) were considered to be HS. In total, 3 581 molecules form the T_CP-_ data set. A complementary T_CP+_ data set consists of the same number of compounds randomly selected from the ZINC15 database [57] that follow the same size distribution as HS compounds and that, in addition, do not exceed any of the aforedescribed complexity indices. Data sets used in the present work are summarized in Fig. 1.

### Performance evaluation

The performance of classification models studied in the present work was assessed by four different metrics: the classification accuracy (*Acc*), sensitivity (*SN*), specificity (*SP*) and area under the ROC curve (*AUC*). *Acc* gives the percentage of correctly classified samples regardless of their class.

8 $${\text{Accuracy }}\left( {\text{Acc}} \right) = \frac{TP + TN}{TP + TN + FN + FP}$$

where true positives (*TP*) are ES compounds predicted by a model to be ES, true negatives (*TN*) are HS compounds predicted to be HS, false positives (*FP*) are HS compounds predicted to be ES and false negatives (*FN*) are ES compounds predicted to be HS. The accuracy can also be evaluated for positive and negative classes independently leading to *SN* and *SP*. *SN* is the percentage of correctly predicted positive class compounds, while the percentage of correctly predicted negative class compounds is known as *SP*.

9 $${\text{Sensitivity }}\left( {\text{SN}} \right) = \frac{TP}{TP + FN}$$

10 $${\text{Specificity }}\left( {\text{SP}} \right) = \frac{TN}{TN + FP}$$

**Fig. 2:**
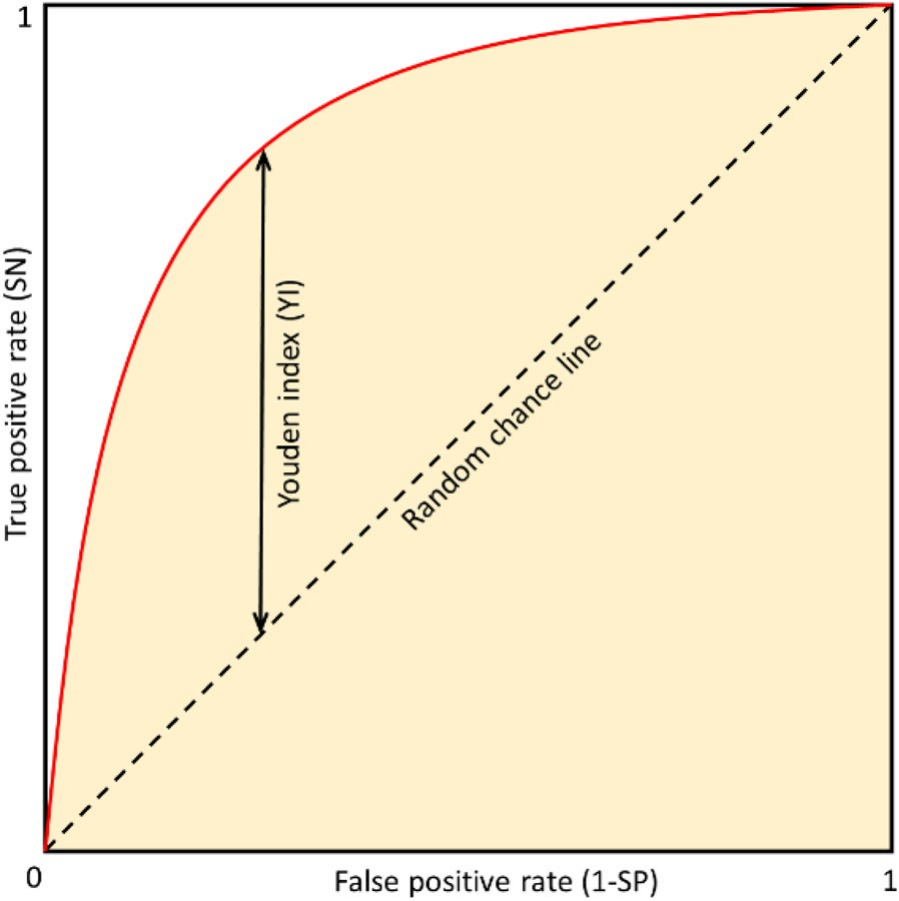
ROC curve and Youden index. The ROC curve (red line) is the dependency of true positive rate (it equals to SN) on false positive rate (it equals to 1-SP) at various thresholds. The random chance line represents a classifier that assigns examples into individual classes randomly. Orange shaded area represents the area under the ROC curve (AUC). The larger the AUC, the better is the overall performance of the classifier. Youden index (YI) is the point on the ROC curve that is farthest from the random chance line along the SN axis

*SN* and *SP* can be combined in the receiver operating characteristic (ROC) curve that is the graphical representation of the trade-off between true positive rate (given as *SN*) and false positive rate (given as 1 − *SP*) over all possible thresholds (Fig. 2). The area under the ROC curve (*AUC*) is the quantitative measure of the performance of a classifier and is equal to the probability that a classifier will rank a randomly chosen positive example higher than a randomly chosen negative example. A random classifier has *AUC* of 0.5, while *AUC* for a perfect classifier is equal to 1.

### Random forest classification, SAScore and SCScore

Because of its wide adoption in various cheminformatics applications [58, 64–66], the random forest (RF) classifier with compounds encoded by 1024-bits long Morgan fingerprint with radius 2 was used as a baseline method with which SYBA, SAScore [45] and SCScore [43] were compared. The RF classifier was implemented in Scikit-learn [67]. Two RF hyperparameters were optimized: the number of trees (50, 100, 300 and 500) and the maximum number of features considered when looking for the best split (10% out of 1024 = 102, 25% = 256, 50% = 512, 75% = 768, 100% = 1024, $\sqrt {1024} = 32$ and $\log_{2} \left( {1024} \right) = 10$). For each pair of hyperparameters, RF model was trained using the training set S and the prediction accuracy was evaluated on the test set T_CP_ (Additional file 2: Table S2, Figures S2–S8). The setting used in this work (100 trees and 32 features) represents the best trade-off between computational efficiency and prediction accuracy [64]. RF was trained using the training set S (Fig. 1). SAScore was calculated by the RDKit toolkit [60]. SCScore code was downloaded from the public GitHub repository [68].

### Classification thresholds

In SYBA, more positive value means a higher probability that the compound is ES and more negative value indicates a higher probability that the compound is HS (Eq. 4). The threshold value of zero is used to distinguish between ES and HS compounds. For SAScore, the recommended value of 6.0 [45] was used as a threshold. In RF, the final prediction is based on a number of decision trees that predict either of classes. Here, 0.5 is used as a threshold, i.e., if more decision trees predict ES than HS class, a compound is classified as ES and vice versa. For SCScore [43], no threshold was suggested by the authors. In such case, the threshold can be identified by the analysis of the ROC curve. A frequently used measure that enables the selection of an optimal threshold is the Youden index (*YI*) [69, 70]. *YI* is defined as

11 $$YI = { \hbox{max} }\left( {SN + SP - 1} \right)$$

and ranges between 0 and 1 (Fig. 2). The optimal threshold value is selected by maximizing *YI,* i.e., by maximizing the sum of *SN* and *SP*.

### Statistical comparison of model performance

The performance of studied classification models was compared using non-parametric Cochran’s Q test [71], an omnibus test for testing for differences between three or more machine learning models. In the case of the statistically significant result of Cochran’s Q test, differing pairs of classification models were identified by McNemar’s post hoc paired test [72] with Benjamini–Hochberg false discovery rate adjustment [73]. McNemar’s test checks if the distribution of disagreements between two methods is imbalanced. The statistical significance for all tests in the present work was assessed at the significance level α = 0.05.

## Results and discussion

### Chemical space covered by SYBA data sets

**Fig. 3:**
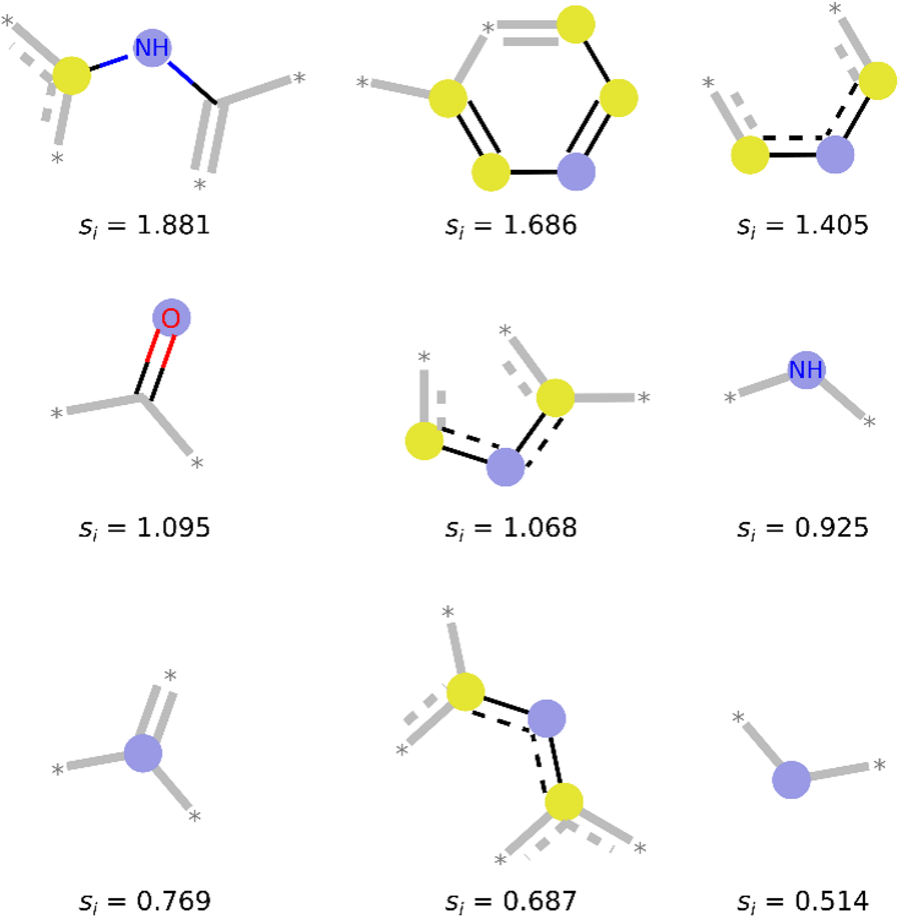
ES fragments enriched in the S_+_ data set. Nine fragments that are most frequent in the S_+_ data set and, at the same time, least frequent in the S_-_ data set. *s*_*i*_ is SYBA fragment score. Blue circles represent each fragment central atom, yellow circles represent aromatic atoms. Fragment images were generated by the RDKit function DrawMorganEnvs()

**Fig. 4:**
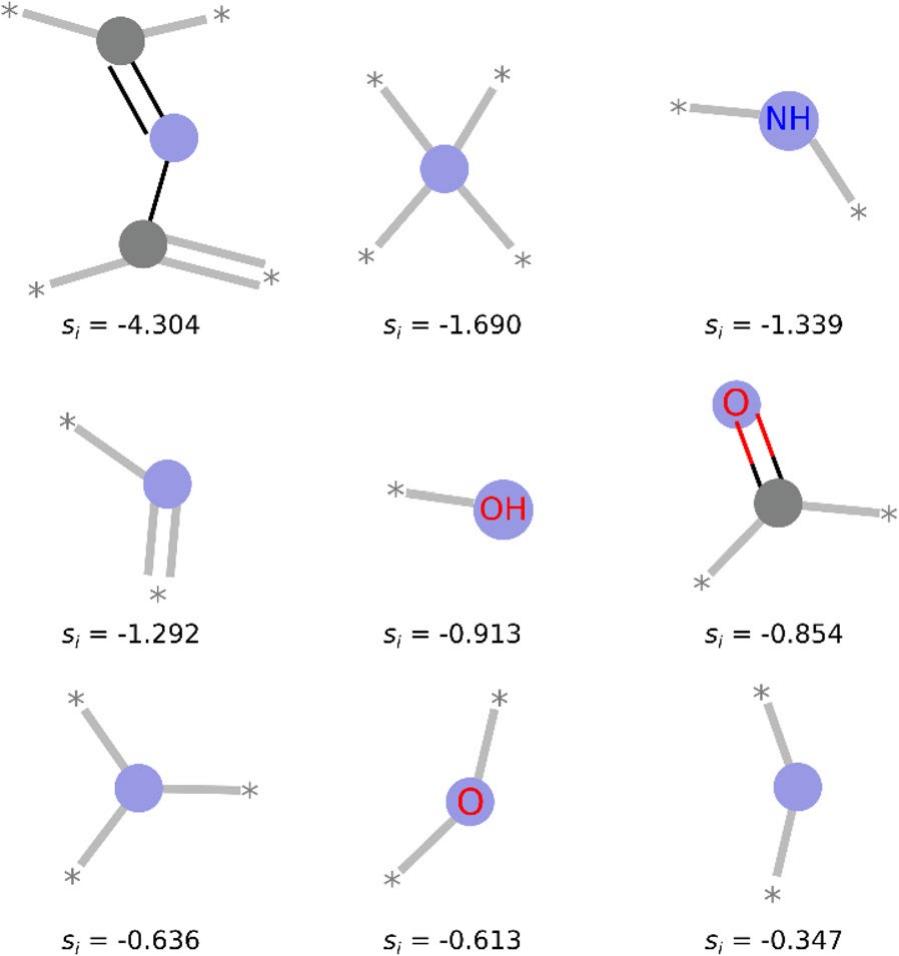
HS fragments enriched in the S- data set. Nine fragments that are most frequent in the S- data set and, at the same time, least frequent in the S_+_ data set. s_i_ is SYBA fragment score. Blue circles represent fragment central atom, gray circles represent aliphatic ring atoms. Fragment images were generated by the RDKit function DrawMorganEnvs()

The examples of training set compounds are given in Additional file 2: Figures S9–S12. In total, 3 439 074 ECFP8 fragments were obtained for ES compounds and 23 447 524 fragments for HS compounds. 458 040 fragments are common for both S + and S- subsets. 55.0% of S+ fragments and 91.7% of S− fragments are present only once in the whole data set S (singletons). Typical ES and HS fragments are shown in Figs. 3 and 4, fragments with very low SYBA in Additional file 2: Figure S13 and compounds containing these fragments in Additional file 2: Figure S14.

**Fig. 5:**
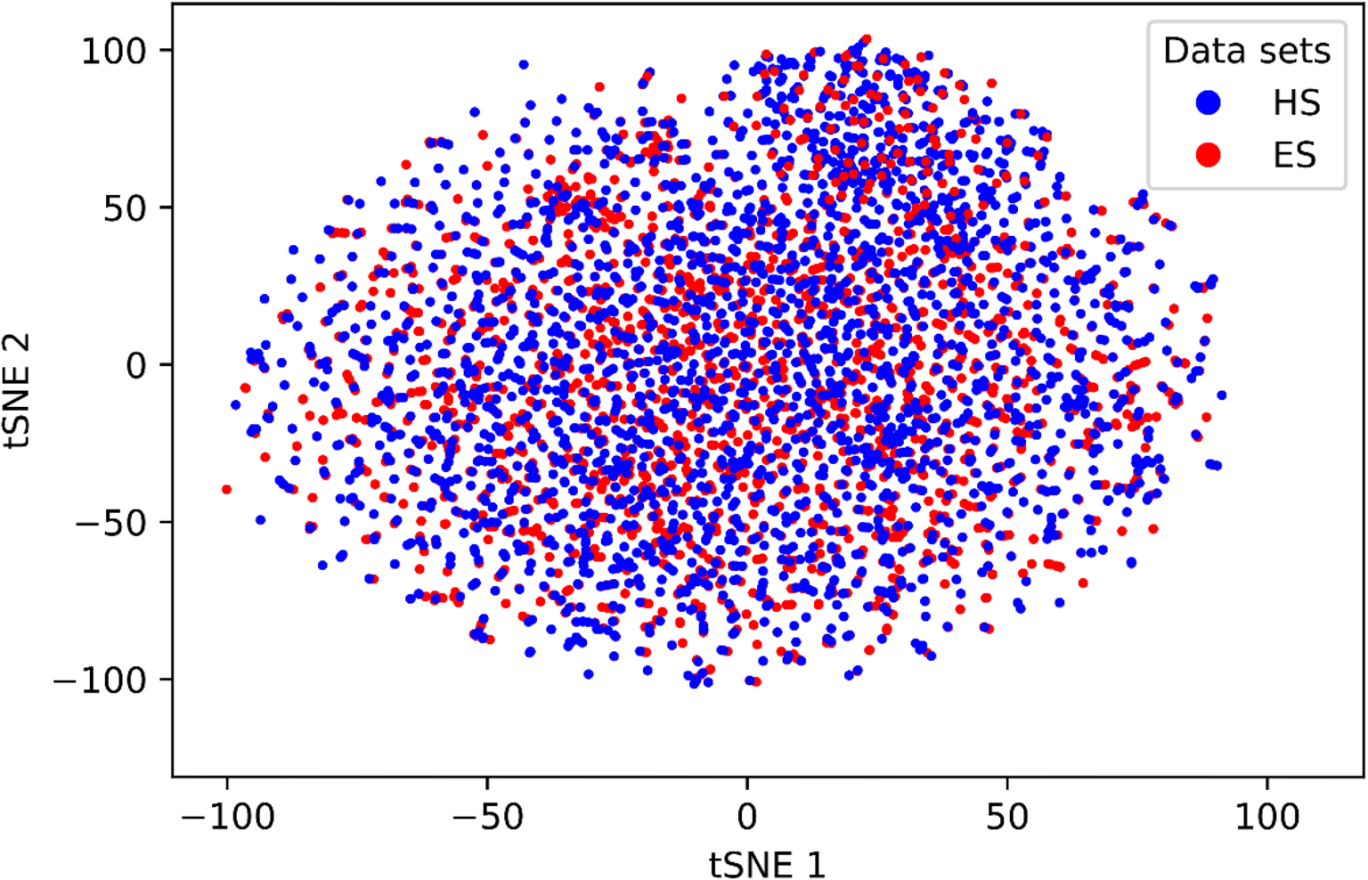
Chemical space coverage by ES and HS training set compounds. 3000 ES and 3000 HS compounds were randomly selected from the training set and each compound was encoded by 1024 bits long ECFP4 fingerprint. The dimensionality of the input space was reduced by SVD to 500 components that explain 85% of the variance in the data

Though the number of fragments in HS compounds is much larger than in ES compounds, chemical space is equally covered by both HS and ES molecules and there is no bias towards HS compounds (Fig. 5).

The visualization of chemical space (Fig. 6) covered by S, T_CP_ and T_MC_ compounds shows that test set compounds lie within chemical space of training compounds. The examples of T_CP_ and T_MC_ compounds are given in Additional file 2: Figures S15–S21.

**Fig. 6:**
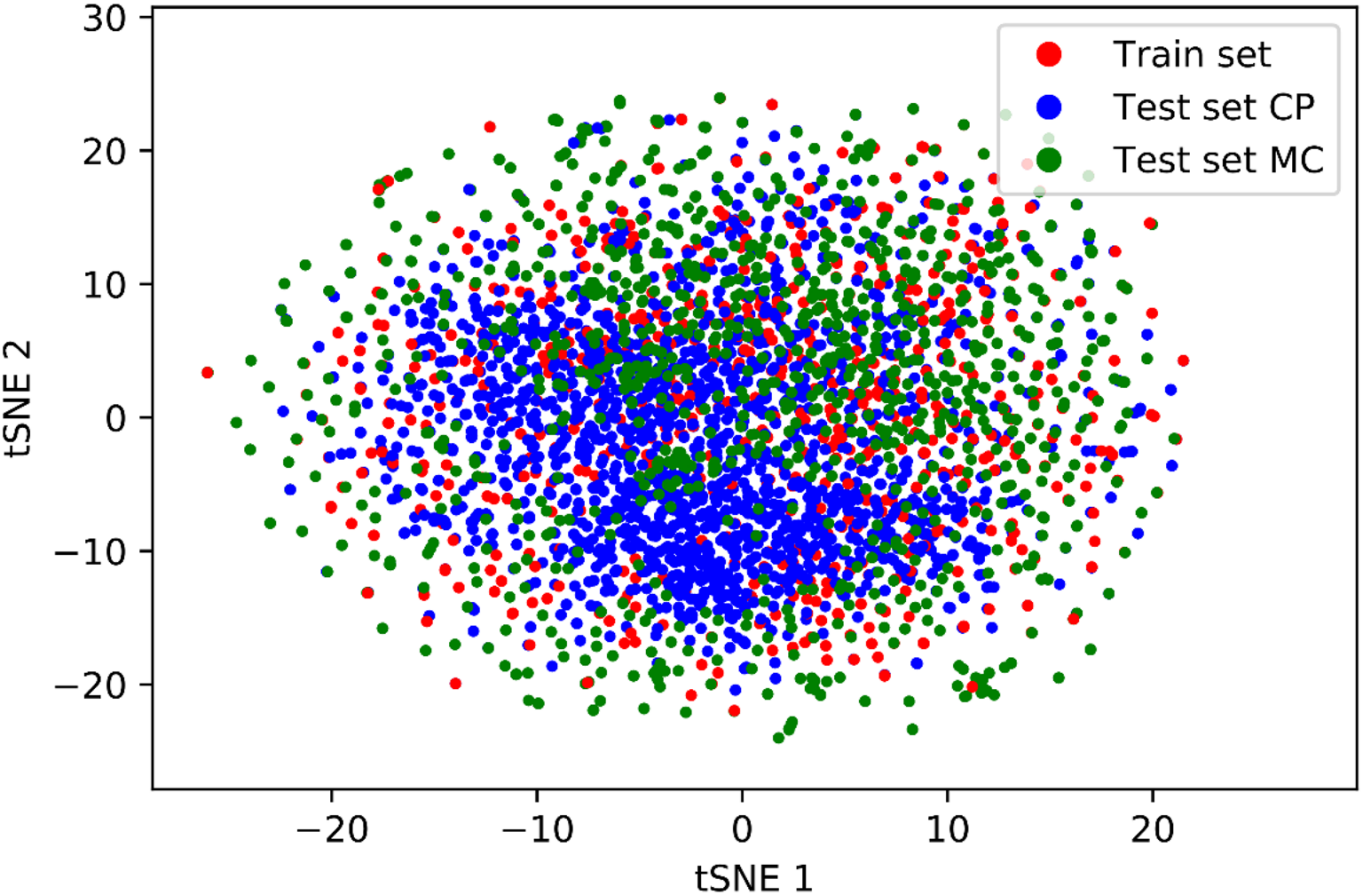
Chemical space coverage by training set S and test sets T_CP_ and T_MC_. T_MC_ data set consists of 40 HS compounds and 1200 ES compounds, from S and T_CP_ data sets random samples of 1240 compounds were generated. Each compound was encoded by 1024 bits long ECFP4 fingerprint. The dimensionality of the input space was reduced by SVD to 500 components that explain 88% of the variance in the data

**Fig. 7:**
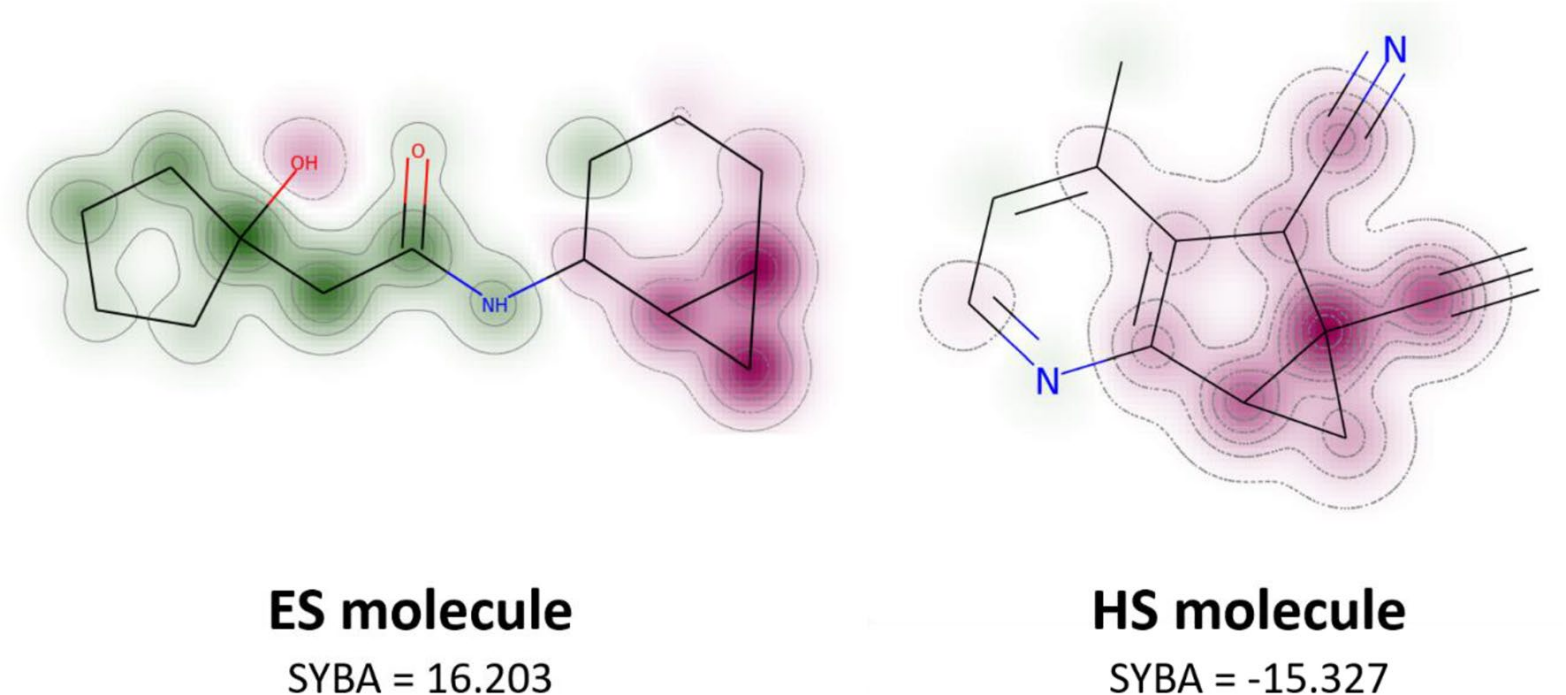
SYBA fragment score visualization. Fragment score is projected on the fragment root atom and the whole molecule is visualized as a similarity map [74]. The more frequent the fragment is in the S_+_ data set compared to the S_-_ data set, the greener is its central atom. Similarly, the more frequent the fragment is in the S_-_ data set compared to the S_+_ data set, the redder is its central atom. This visualization enables to analyze the contributions of the individual parts of the molecule to its synthetic accessibility. In the HS molecule, the quaternary carbon is most problematic. Another substructure decreasing compound synthetic accessibility is a fused cyclopropane ring as can be observed both in ES and HS compounds

SYBA also enables the visualization and interpretation of fragment score contributions. Each SYBA fragment score can be projected to the corresponding fragment root atom and this projection can be used to analyze which fragments contribute unfavorably to molecule synthetic accessibility (Fig. 7).

### Classifier performance on manually curated test set

The differences in classification of T_MC_ (Fig. 1) compounds using SYBA, SAScore and RF with default thresholds are statistically significant. The Cochran’s Q test *p* value is 2 × 10^−5^ for the T_MC_ test set with the smallest SYBA AUC. The results of the corresponding McNemar’s paired tests [72] are summarized in Table 1. While RF and SYBA do not differ significantly, both RF and SYBA yield significantly better results than SAScore.

**Table 1:** The results of McNemar’s two-sided paired tests for the T_MC_ test set with the smallest SYBA AUC (AUC = 0.830)

	Adjusted p-value
RF vs. SAScore	0.002
RF vs. SYBA	1.000
SAScore vs. SYBA	0.001

The default threshold values were used (0.0 for SYBA, 0.5 for RF, and 6.0 for SAScore). The p-values were adjusted using Benjamini–Hochberg method

**Fig. 8:**
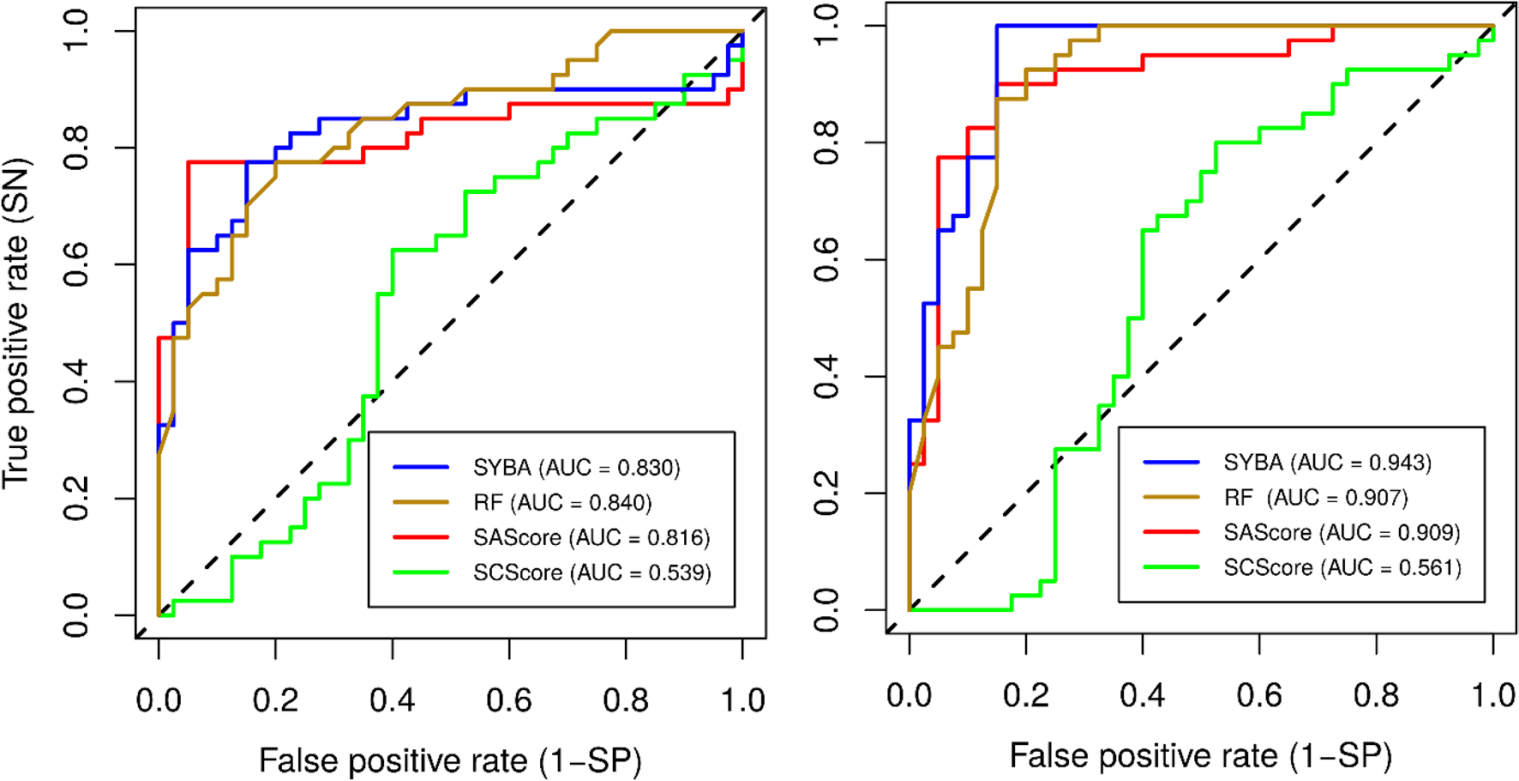
The ROC curves of classification models for the manually curated T_MC_ test set. Out of 30 possible T_MC_ instances, ROC curves of the T_MC_ test set with the smallest (left) and largest (right) SYBA AUC are shown

The quality measures of the classification of the compounds in the manually curated T_MC_ test set, averaged over 30 T_MC_ instances, are summarized in Table 2. The corresponding confusion matrices are reported in Additional file 2: Panel S1 and Panel S2 and ROC curves are shown in Fig. 8.

**Table 2:** The performance of classification models for the manually curated T_MC_ test set

Model	*AUC*	*Acc*	*SN*	*SP*	Threshold
Default threshold					
SYBA	*0.903*	*0.844*	0.913	*0.775*	0.0
SAScore	0.865	0.617	*0.934*	0.300	6.0
RF	0.875	0.819	0.863	0.775	0.5
Optimized threshold					
SYBA	*0.903*	*0.871*	*0.902*	0.840	19.1
SAScore	0.865	0.859	0.799	*0.919*	3.9
SCScore	0.528	0.601	0.707	0.496	3.7
RF	0.875	0.842	0.855	0.828	0.6

Quality measures *AUC*, *Acc*, *SN* and *SP*, as well as thresholds, are reported as their average values over 30 T_MC_ instances

In terms of *Acc*, the best performing model is SYBA followed by RF and SAScore. While SYBA and RF sensitivity and specificity are well balanced, SAScore shows high sensitivity (*SN* = 0.934, i.e., on average 93.4% of *ES* compounds are predicted as *ES*), while its specificity (i.e., the ability to correctly classify *HS* compounds) is rather low (*SP* = 0.300). The observed high sensitivity of SAScore is not surprising as only 0.2% of ZINC structures have SAScore greater than 6.0 and out of these, only lower units were selected into the T_MC+_ set.

For optimized thresholds, the differences between SYBA, SAScore and RF are again statistically significant (Cochran’s Q test p-value is 2 × 10^−14^ for the T_MC_ test set with the smallest SYBA AUC). However, McNemar’s paired test (Table 3) identifies significant differences only between SCScore and other methods meaning that SAScore results improve significantly upon threshold optimization.

**Table 3:** The results of McNemar’s two-sided paired tests for the T_MC_ test set with the smallest SYBA AUC (AUC = 0.830)

	Adjusted p-value
RF vs. SAScore	0.164
RF vs. SCScore	2 × 10^−6^
RF vs. SYBA	0.687
SAScore vs. SCScore	1 × 10^−8^
SAScore vs. SYBA	0.466
SCScore vs. SYBA	2 × 10^−6^

The optimized threshold values were used (27.7 for SYBA, 0.5 for RF, 3.7 for SAScore, and 4.0 for SCScore). The p-values were adjusted using Benjamini–Hochberg method

In terms of performance measures (Table 2), the most accurate classifiers are SYBA, RF and SAScore followed afar by SCScore. Notable is the improvement of SAScore *SP* by 0.619 compared to the default threshold. The increase in SAScore *SP* comes, however, at the cost of *SN* that decreases by 0.135. The worst performing model, SCScore, is only slightly better (*AUC* = 0.528) than a random model. However, because T_MC+_ and T_MC−_ data sets consist of only 40 compounds each, the results must be interpreted with caution as small changes in confusion matrices lead to relatively large changes in reported metrics.

### Classifier performance on computationally picked test set

Even stronger evidence of the differences between the models is provided by the classification of compounds in the large computationally picked T_CP_ test set (Fig. 1). Using the default thresholds, the differences between the classifiers are statistically significant (Cochran’s Q test p-value < 10^−16^) and all classifiers differ significantly (Table 4).

**Table 4:** The results of McNemar’s two-sided paired tests for the T_CP_ test set

	Adjusted p-value
RF vs. SAScore	< 10^−16^
RF vs. SYBA	< 10^−16^
SAScore vs. SYBA	< 10^−16^

The default threshold values were used (0.0 for SYBA, 0.5 for RF, and 6.0 for SAScore). The p-values were adjusted using Benjamini–Hochberg method

When used with their default thresholds, both SYBA and RF are more accurate than SAScore (Table 5, Additional file 2: Panel S3). Low observed SAScore accuracy (*Acc* = 0.665) is caused by its low specificity when almost 70% of HS compounds are predicted to be ES (Table 5).

**Table 5:** The performance of classification models for the computationally picked T_CP_ test set

Model	*AUC*	*Acc*	*SN*	*SP*	Threshold
Default threshold					
SYBA	0.903	*0.962*	0.925	*1.000*	0.0
SAScore	*0.999*	0.665	*0.999*	0.331	6.0
RF	0.995	0.892	0.784	0.999	0.5
Optimized threshold					
SYBA	0.998	0.988	0.985	0.991	− 18.6
SAScore	*0.999*	*0.990*	*0.986*	*0.993*	4.5
SCScore	0.641	0.612	0.499	0.725	3.1
RF	0.995	0.973	0.960	0.986	0.2

Cochran’s Q test identifies significant differences (p-value < 10^−16^) also if classifiers are used with the optimized thresholds. While statistically significant differences were detected within RF/SAScore and RF/SYBA pairs (Table 6), the effect size is still rather small (Table 5). However, the difference between SCScore and all other methods is statistically significant and large (Tables 5 and  6). No statistically significant difference was observed between SYBA and SAScore meaning that these two methods yield comparable results.

**Table 6:** The results of McNemar’s two-sided paired tests for the T_CP_ test set

	Adjusted p-value
RF vs. SAScore	4 × 10^−15^
RF vs. SCScore	< 10^−16^
RF vs. SYBA	2 × 10^−14^
SAScore vs. SCScore	< 10^−16^
SAScore vs. SYBA	0.474
SCScore vs. SYBA	< 10^−16^

The optimized values of threshold (− 18.6 for SYBA, 0.2 for RF, 4.5 for SAScore, 3.1 for SCScore) were used. p-values were adjusted using Benjamini–Hochberg method

Compared to its default threshold of 6.0, SAScore specificity increases by 0.662 (Table 5) when the threshold is shifted to the optimal value of 4.5 (Fig. 10). At this threshold, SAScore is on par with SYBA and RF methods (Table 5). However, SYBA retains its high performance over much broader range of threshold values than SAScore (Additional file 2: Figure S1).

**Fig. 9:**
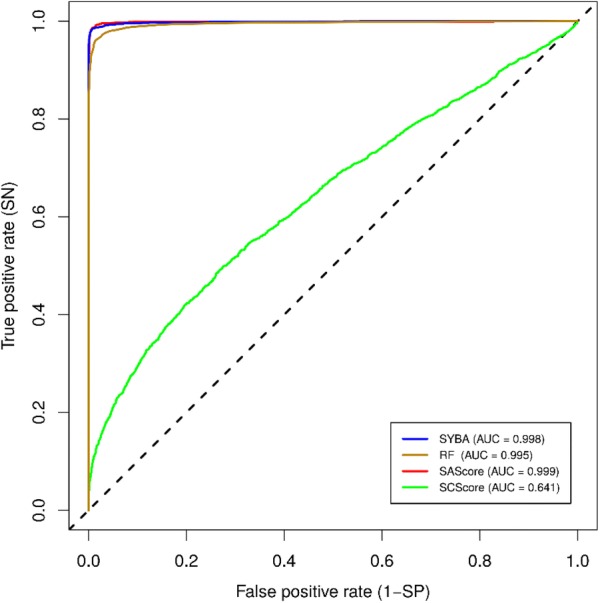
ROC curves of classification models for the T_CP_ test set

**Fig. 10:**
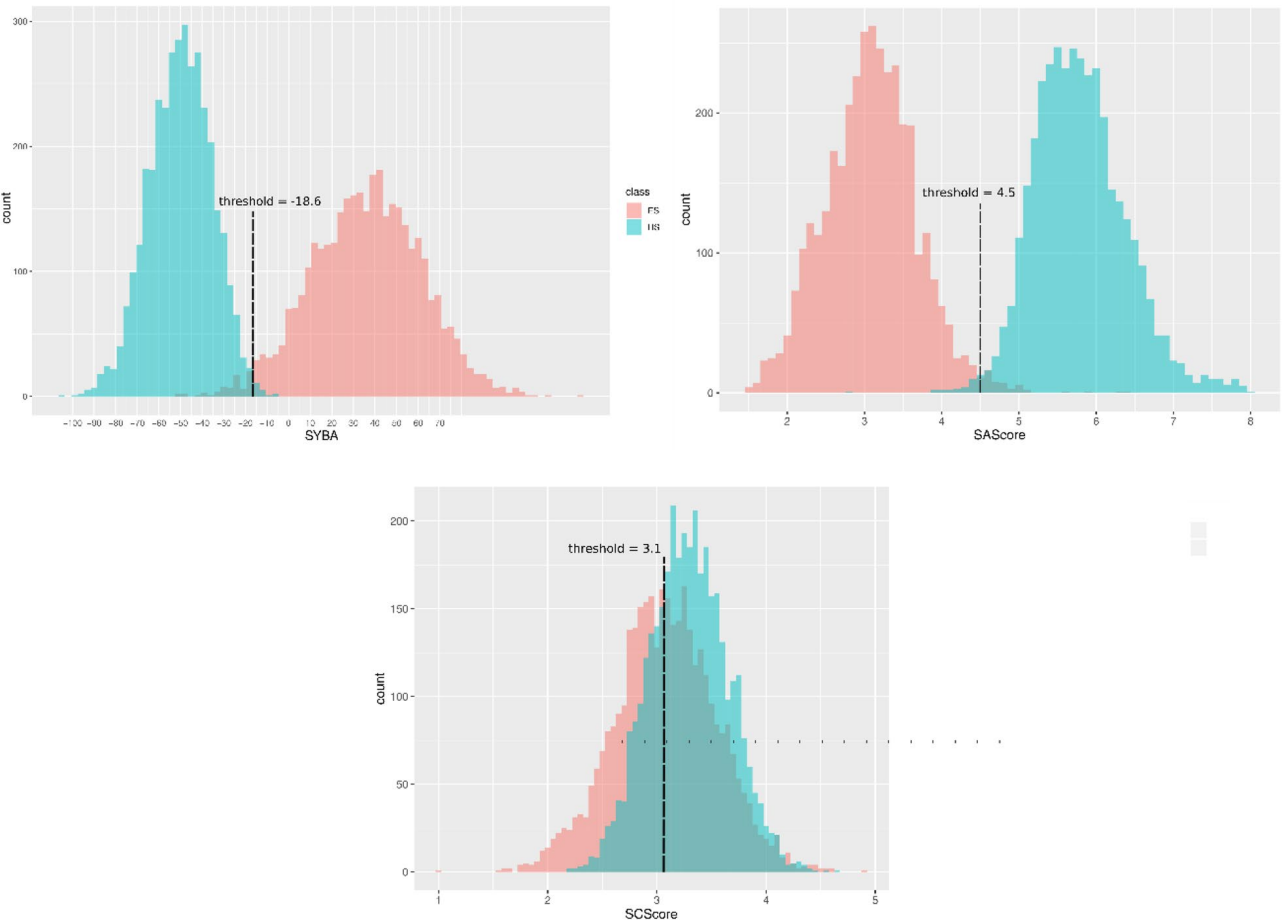
SYBA, SAScore and SCScore histograms of ES and HS compounds in the computationally picked T_CP_ test set. The positions of optimal thresholds are shown. SAScore recommended threshold of 6.0 leads to a large number of FP (Additional file 2: Panel S3). If the threshold is moved to its optimal value of 4.5, SAScore specificity increases from 0.317 to 0.994, i.e. by 0.677

High performance of SYBA, RF and SAScore is also evident from their *AUC* that is close to one (Fig. 9, Table 5). On the other hand, SCScore fails to distinguish between ES and HS compounds as can be deduced from its ROC curve (Fig. 9). In its optimal threshold of 3.1, SCScore predicts a majority of T_CP_ compounds as HS (Fig. 10, Additional file 2: Panel S3).

**Fig. 11:**
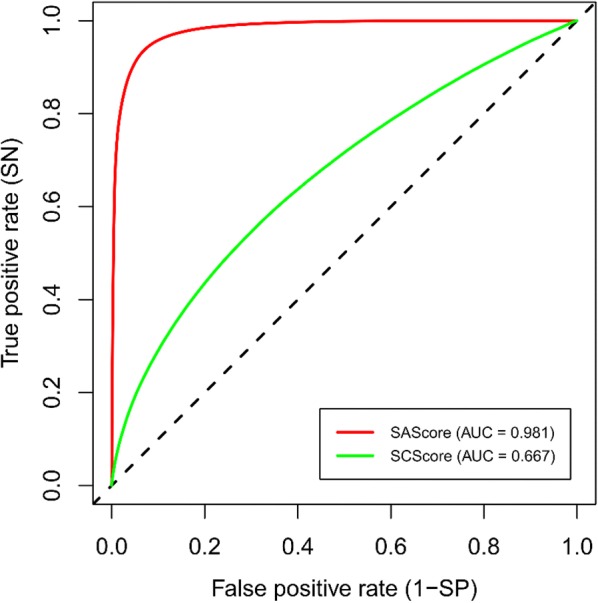
ROC curves of classification models for the training set S

In addition to the T_MC_ and T_CP_ test sets, the performance of SAScore and SCScore was also assessed using the training set S, as this data set was not used for their parametrization. Classification results are shown in Table 7 and Fig. 11, confusion matrices are available in Additional file 2: Panel S4.

**Table 7:** The performance of classification models for the training set S

Model	*AUC*	*Acc*	*SN*	*SP*	Threshold
Default threshold					
SAScore	0.981	0.767	0.998	0.536	6.0
Optimized threshold					
SAScore	*0.981*	*0.933*	*0.935*	*0.932*	4.4
SCScore	0.667	0.623	0.564	0.682	3.7

In agreement with previous experiments on the T_MC_ and T_CP_ test sets, SAScore is able to distinguish between ES and HS compounds more accurately than SCScore. However, to achieve the best performance, SAScore classification threshold must be shifted from its default value of 6.0 to the optimal value of 4.4. At this threshold, SAScore is both highly sensitive and specific, while SCScore is, using its optimal threshold of 3.7, sensitive and specific only moderately.

The observed poor performance of SCScore in all data sets may follow from the fact that SCScore differs conceptually from other methods tested in the present work. In SCScore, the problem of predicting synthetic complexity is reformulated as the analysis of reactions consisting of reactant-product pairs and SCScore correlates with a number of reaction steps. It means that, contrary to SYBA and SAScore, SCScore captures synthetic feasibility of compounds, not structural complexity. In SCScore derivation, each molecule is analyzed as a whole in the context of all molecules and reactions as they appear in the Reaxys database [44]. Thus, SCScore is biased [43] by the types of reactants and products in the Reaxys database. Therefore, we hypothesize that the unsatisfactory results of SCScore are caused by the fact that compounds in our data sets come from chemical subspace insufficiently covered by Reaxys compounds.

## Conclusions

In the present work, SYBA method for the classification of organic compounds as easy- and hard-to-synthesize is described. SYBA is an additive fragment-based approach meaning that the compound is decomposed into individual substructure fragments, each fragment is assigned its respective SYBA fragment score and these are summed to obtain the final SYBA score. The fragment scores were derived by the Bayesian analysis of the frequency of ECFP8 fragments occurring in the database of ES compounds, that were randomly chosen from the ZINC15 database [57], and HS compounds, that were generated using the Nonpher approach [58]. Because SYBA was derived from ECFP8 fragments that utilize only molecular connectivity and no 3D information, the influence of stereochemistry on synthetic accessibility is not accounted for. However, apart from ECFP8 fragments, the number of stereocenters is included in SYBA calculation and the compounds with many stereocenters are penalized. If the SYBA score is positive, the compound is considered to be ES and vice versa. While SYBA score can theoretically assume values between plus and minus infinity, a majority of compounds will have SYBA score between − 100 and +100 in real applications. It must be stressed here that the absolute value of the SYBA score is the measure of the confidence of the prediction and not of the degree of the synthetic accessibility.

SYBA was compared with other two recent classification methods, SAScore [45] and SCScore [43]. As a baseline for the comparison, RF/ECFP4 classifier was used due to its wide adoption in many cheminformatics applications. All methods were assessed using accuracy, sensitivity, specificity and area under the ROC curve. While SYBA and RF provide similar performance, we recommend to use SYBA due to its smaller complexity, lower computational demands and more straightforward analysis of the individual fragment contributions. SYBA outperforms SAScore when this is used with the threshold of 6.0 proposed by the authors [45]. However, if the SAScore threshold is changed to the value of ~ 4.5, the accuracy of SYBA and SAScore becomes comparable. Therefore, to reduce the number of false positives in workflows that utilize SAScore, we recommend to decrease the SAScore threshold to ~ 4.5. SYBA, RF and SAScore substantially outperform SCScore. The observed weak performance of SCScore can be, in our opinion, attributed to the fact that our test set compounds come from a part of chemical space that is insufficiently covered by Reaxys compounds used to derive SCScore. The SYBA fragment scores can be mapped [74] onto a molecule and used for the analysis of the contribution of its individual substructures to the overall synthetic accessibility.

SYBA can be used to quickly rank large molecular data sets that originate, for example, from de novo molecular design. However, SYBA is conceptually based on the notion that a compound can be categorized as easy- and hard-to-synthesize. As the synthetic accessibility is a vaguely defined term, SYBA’s simplifying approach, though accurate enough, cannot compete with more sophisticated synthetic path-reconstruction methods that enable the incorporation of other factors such as the availability of starting materials, reaction yields or a price aspect. In the end, the definitive assessment of synthetic accessibility is in the hands of experienced organic chemists.

## Supplementary information

**Additional file 1.** Training set S. It consists of 693 353 ES compounds selected from the ZINC15 database and of 693 353 ES compounds generated by Nonpher.

**Additional file 2.** This supporting document contains the detailed description of the SYBA score derivation, threshold values of complexity indices, RF hyperparameter optimization results, the dependence of the accuracy of the classification of TCP compounds on SYBA and SAScore thresholds, examples of correctly predicted and mispredicted S, TMC and TCP compounds, fragments with very low SYBA contributions and HS compounds containing these fragments, and confusion matrices of the classification of TMC, TCP and S data sets.

**Additional file 3.** Manually curated test set (TMC). It consists of 40 HS compounds manually selected from scientific papers and of 30 ES sets, each of them contains 40 compounds selected from the ZINC15 database.

**Additional file 4.** Computationally picked test set (TCP). It consists of 3 581 HS compounds that were obtained from the GDB-17 database complemented by the same number of compounds randomly selected from the ZINC15 database.

## Data Availability

The code used to train and benchmark SYBA model is available from https://github.com/lich-uct/syba repository. Nonpher is available from https://github.com/lich-uct/nonpher.

## Abbreviations

Acc: Accuracy
AUC: Area under the ROC curve
ES: Easy-to-synthesize
HS: Hard-to-synthesize
MW: Molecular weight
RF: Random forest
ROC: Receiver operating characteristic
S: Training set
SN: Sensitivity
SP: Specificity
SVD: Singular value decomposition
SYBA: SYnthetic Bayesian Accessibility
T_CP_: Computationally picked test set
T_MC_: Manually curated test set
YI: Youden index
