# Supplementary material for: SYBA: Bayesian estimation of synthetic accessibility of organic compounds
Source: J Cheminform. 2020 May 20;12:35. doi: 10.1186/s13321-020-00439-2 (PMC7238540; doi:10.1186/s13321-020-00439-2)
Supplement: Supplementary file 2 — Additional file 2. This supporting document contains the detailed description of the SYBA score derivation, threshold values of complexity indices, RF hyperparameter optimization results, the dependence of the accuracy of the classification of TCP compounds on SYBA and SAScore thresholds, examples of correctly predicted and mispredicted S, TMC and TCP compounds, fragments with very low SYBA contributions and HS compounds containing these fragments, and confusion matrices of the classification of TMC, TCP and S data sets. [file 13321_2020_439_MOESM2_ESM.docx]

## SYBA score derivation

In our model, we distinguish between the presence and absence of the fragment *i* in the molecule, which is expressed by $f_{i}=1$, resp. $f_{i}=0$. The collection of $f_{i}$ values for all compounds of some class $C$ forms a finite sequence of binary random variables $f_{i}$ and can be modeled by a Bernoulli process. This Bernoulli process is parametrized by the probability $w_{C, i}$ to find the fragment *i* in the molecule belonging to the class $C$. This parameter expresses our prior knowledge that the molecule belongs to the class $C$ and it is a random variable per se. Therefore, it holds

Equation S1 $p\left( f_{i} | C \right)= \int_{0}^{1} p\left( f_{i} | w_{C, i}, C \right) p(w_{C, i}| C)dw_{C, i}.$

Here, the prior $p(w_{C,i}|C)$ represents our knowledge that molecules from the class $C$ contain the fragment $i$. The prior $p(w_{C,i}|C)$ is estimated from the training set S consisting of $N_{C}$ molecules belonging to the class $C$, $n_{C,i}$ of which contain the fragment *i*. In SYBA, a Bernoulli process conjugate prior, that enables analytic solution, is used. It can be expressed as the beta distribution

Equation S2 $p\left( w_{C,i} | C \right)=\frac{1}{B\left( n_{C,i}+1, N_{C}-n_{C,i}+1 \right)}{w_{C,i}}^{n_{C,i}}{(1-w_{C,i})}^{N_{C}-n_{C,i}}$,

where B is the Euler beta function. The likelihood $p\left( f_{i} | w_{C, i}, C \right)$ in Equation S1 can be expressed as $p\left( f_{i} | w_{C, i}, C \right)= {w_{C,i}}^{f_{i}}{(1-w_{C,i})}^{1-f_{i}}$. Thus, the posterior $p\left( f_{i} | C \right)$ in Equation S1 can be evaluated analytically using the beta function algebra:

Equation S3 $P\left( f_{i} | C \right)=\int_{0}^{1} \frac{1}{B\left( n_{C,i}+1,N_{C}-n_{C,i}+1 \right)}{w_{C,i}}^{n_{C,i}+f_{i}}\left( 1-w_{C,i} \right)^{N_{C}+1-n_{C,i}-f_{i}}dw_{C,i}=$

$$\frac{B\left( n_{C,i}+f_{i}+1,N_{C}+1-n_{C,i}-f_{i}+1 \right)}{B\left( n_{C,i}+1,N_{C}-n_{C,i}+1 \right)}$$

Since all variables are integers, Equation S3 can be rewritten in a factorial form and further simplified to

Equation S4 $p\left( f_{i} | C \right)=\frac{1}{N_{C}+2}{(n_{C,i}+1)}^{f_{i}}{(N_{C}-n_{C,i}+1)}^{1-f_{i}}$.

Plugging this estimate to the equation for $s_{i}(f_{i})$ (Equation 6 of the main text) yields

Equation S5 $s_{i}\left( f_{i} \right)=\ln\frac{N_{\mathrm{HS}}+2}{N_{\mathrm{ES}}+2} +f_{i}\ln\frac{\left( n_{\mathrm{ES},i}+1 \right)}{\left( n_{\mathrm{HS},i}+1 \right)}{+ (1-f}_{i}) \ln\frac{(N_{\mathrm{ES}}-n_{\mathrm{ES},i}+1)}{\left( N_{\mathrm{HS}}-n_{\mathrm{HS},i}+1 \right)}$,

which is Equation 7 of the main text. When $N_{\mathrm{HS}}=N_{\mathrm{ES}}$, the first term in Equation S5 becomes zero.

| Complexity index | bin (Da) | | | | | | | | | | |
| --- | --- | --- | --- | --- | --- | --- | --- | --- | --- | --- | --- |
|  | <150 | <200 | <250 | <300 | <350 | <400 | <450 | <500 | <550 | <600 | >600 |
| Bertz | 390 | 520 | 673 | 845 | 1043 | 1230 | 1456 | 1644 | 1784 | 2052 | 2540 |
| Barone | 281 | 360 | 438 | 522 | 607 | 695 | 795 | 884 | 968 | 1085 | 1408 |
| Whitlock | 19 | 22 | 25 | 28 | 32 | 36 | 42 | 47 | 51 | 64 | 81 |
| SMCM | 21.7 | 33.7 | 40.1 | 46.2 | 49.5 | 55.2 | 63.5 | 70.0 | 81.3 | 100.7 | 128.9 |

Table S1 Threshold values of complexity indices for various MW bins. The threshold value was determined as 999^th^ permiles of complexity index distribution in 22 723 223 ZINC compounds. For example, 99.9% ZINC compounds with MW between 150 Da and 200 Da have Bertz index less than 520.

|  | Min | Max | sqrt,100 |
| --- | --- | --- | --- |
| AUC | 0.978 (100%,50) | 0.996 (sqrt,500) | 0.996 |
| Acc | 0.945 (100%,500) | 0.974 (log2,500) | 0.974 |
| SN | 0.920 (100%,50) | 0.974 (10%,500) | 0.967 |
| SP | 0.965 (100%,500) | 0.984 (50%,500) | 0.983 |

Table S2 RF classification of T_CP_ data set. AUC, Acc, SN and SP were obtained for varying number of features (log2(1024)=10, sqrt(1024)=32, 10%=102, 25%=256, 50%=512, 75%=768, 100%=1024) and number of trees (50, 100, 300, 500). Displayed are their minimum abd maximum values together with corresponding settings, as well as the results for the setting used in the present manuscript (sqrt(1024) and 100 trees).


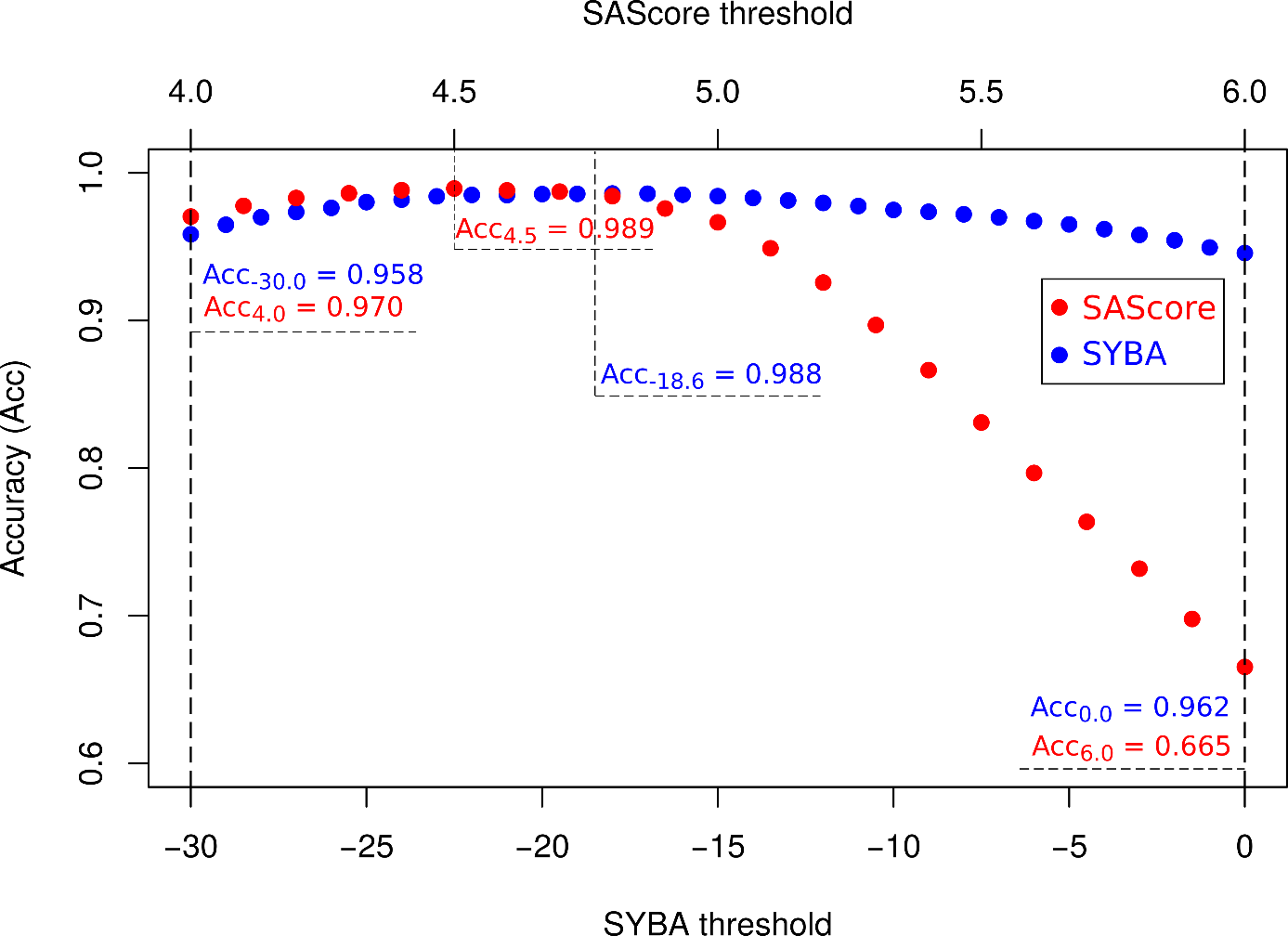


Figure S1 The dependence of the accuracy of the classification of T_CP_ compounds on SYBA and SAScore thresholds. Shown threshold intervals (from -30.0 to 0.0 for SYBA and from 4.0 to 6.0 for SAScore) cover roughly 20 % of all plausible values of SYBA (from -100 to 70) and SAScore (from 1 to 10). Ends of shown threshold intervals are given as implicit thresholds of SYBA (0.0) and SAScore (6.0). Over their respective threshold intervals, SYBA is more robust than SAScore. While for SYBA the difference in the accuracy between optimal and recommended thresholds is only 0.04, this difference equals to 0.324 for SAScore.


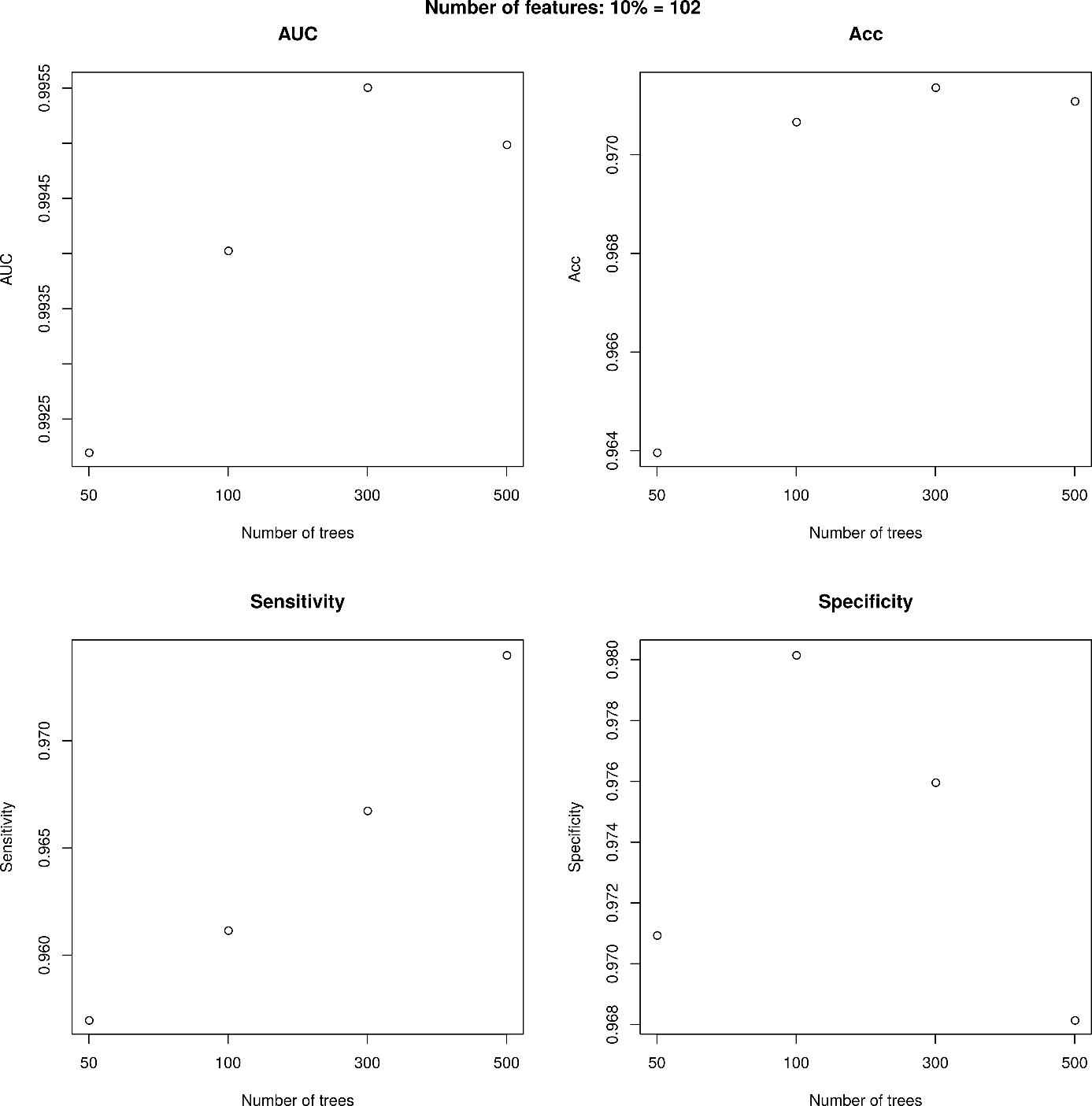


**Figure S2** The dependence of T_CP_ AUC, Acc, SN and SP on the number of trees in the RF (n_estimators keyword in sklearn.ensemble.RandomForestClassifier) for the number of features considered when looking for the best split (max_features keyword) equaling to 10% out of 1024, i.e. for 102 features.


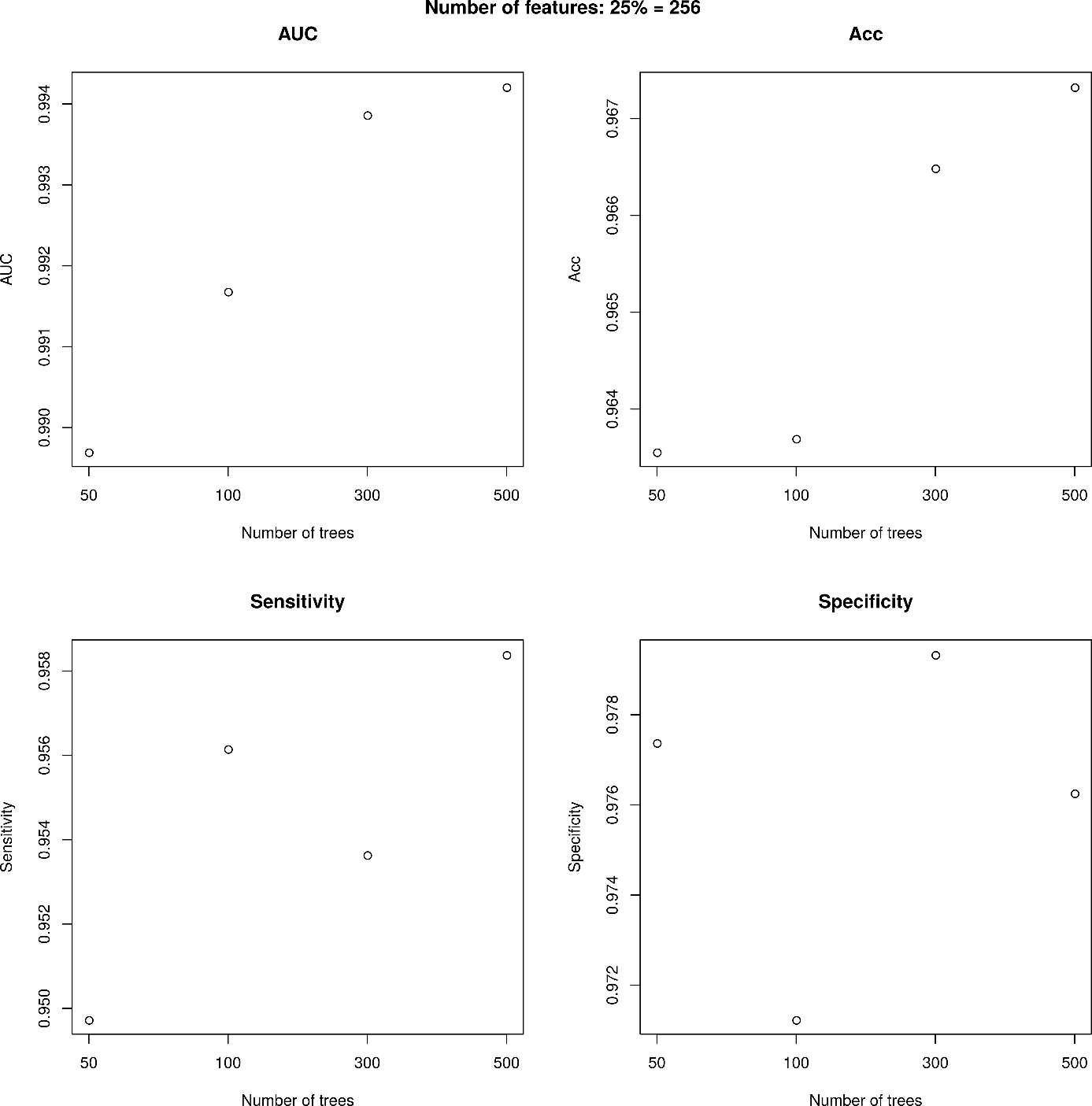


**Figure S3** The dependence of T_CP_ AUC, Acc, SN and SP on the number of trees in the RF (n_estimators keyword in sklearn.ensemble.RandomForestClassifier) for the number of features considered when looking for the best split (max_features keyword) equaling to 25% out of 1024, i.e. for 256 features.


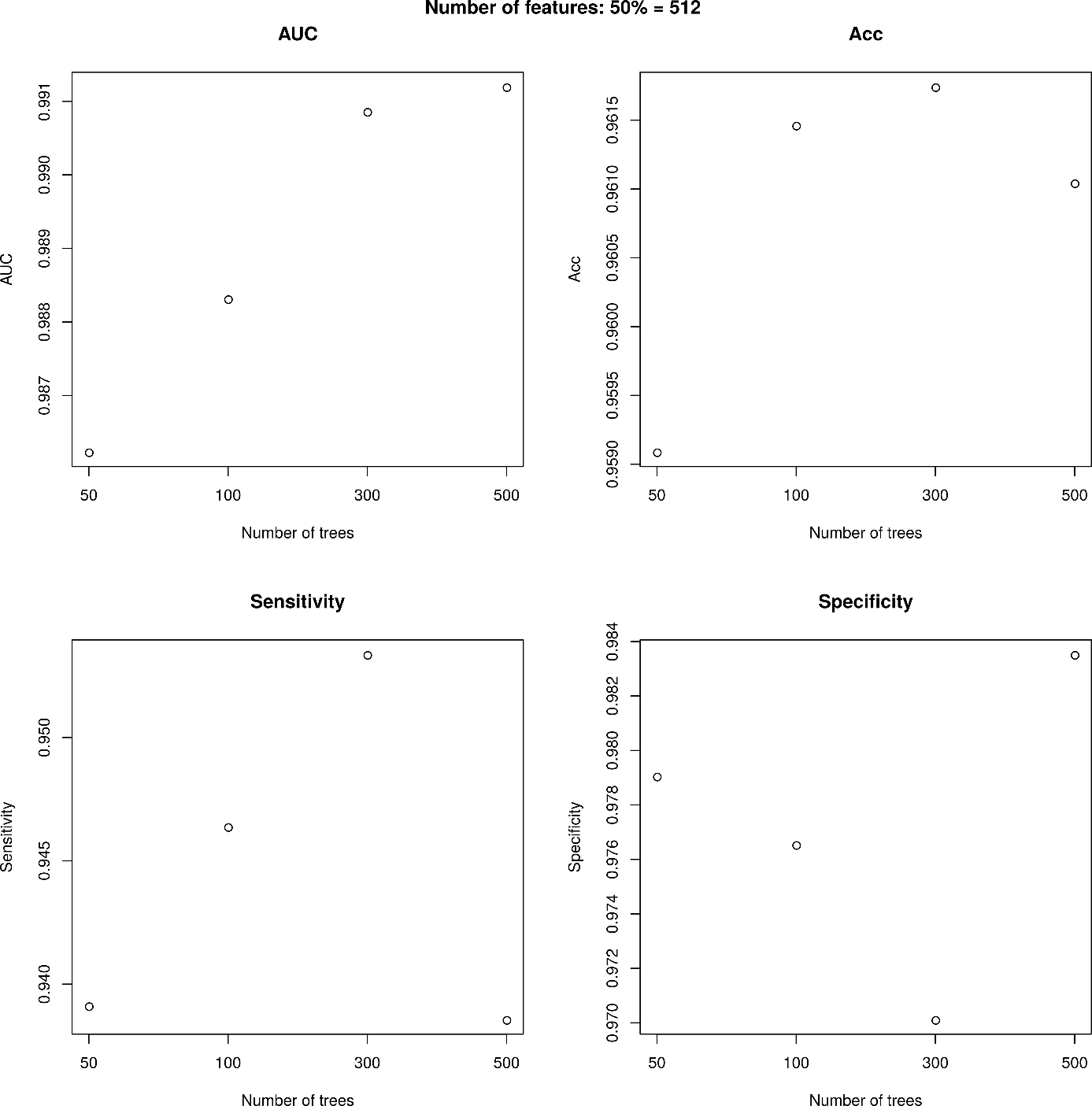


**Figure S4** The dependence of T_CP_ AUC, Acc, SN and SP on the number of trees in the RF (n_estimators keyword in sklearn.ensemble.RandomForestClassifier) for the number of features considered when looking for the best split (max_features keyword) equaling to 50% out of 1024, i.e. for 512 features.


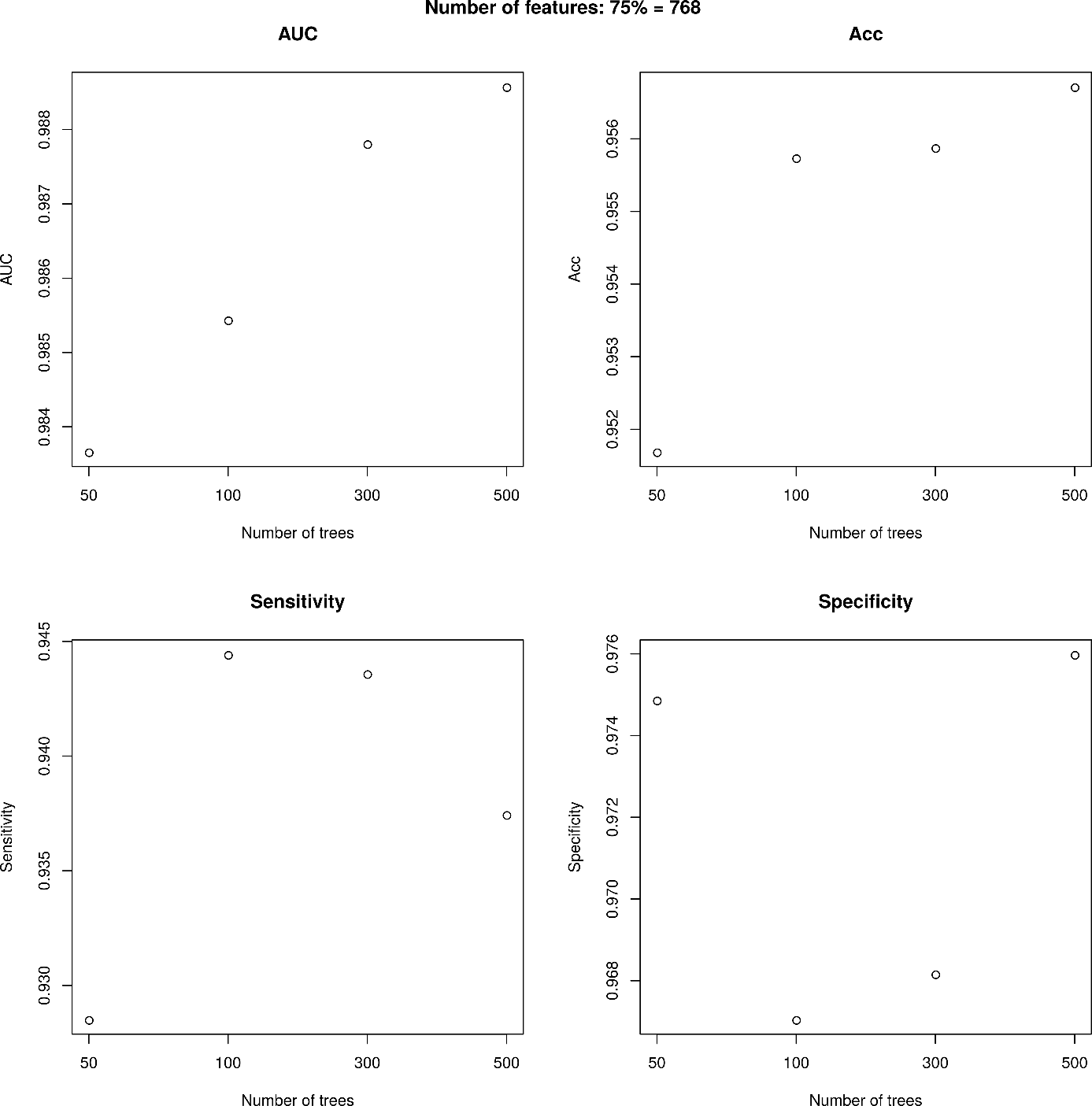


**Figure S5** The dependence of T_CP_ AUC, Acc, SN and SP on the number of trees in the RF (n_estimators keyword in sklearn.ensemble.RandomForestClassifier) for the number of features considered when looking for the best split (max_features keyword) equaling to 75% out of 1024, i.e. for 768 features.


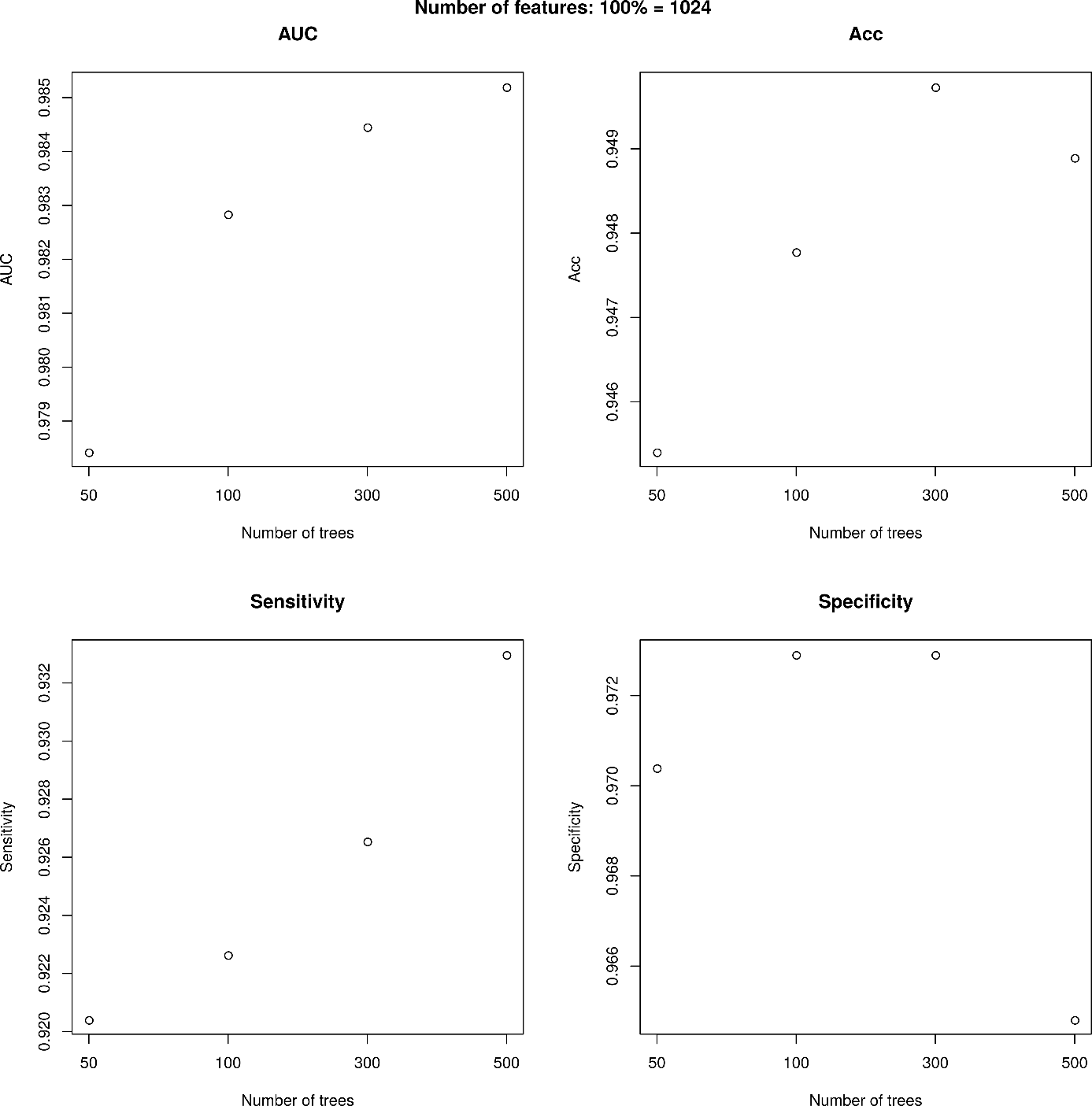


**Figure S6** The dependence of T_CP_ AUC, Acc, SN and SP on the number of trees in the RF (n_estimators keyword in sklearn.ensemble.RandomForestClassifier) for the number of features considered when looking for the best split (max_features keyword) equaling to 100% out of 1024, i.e. for 1024 features.


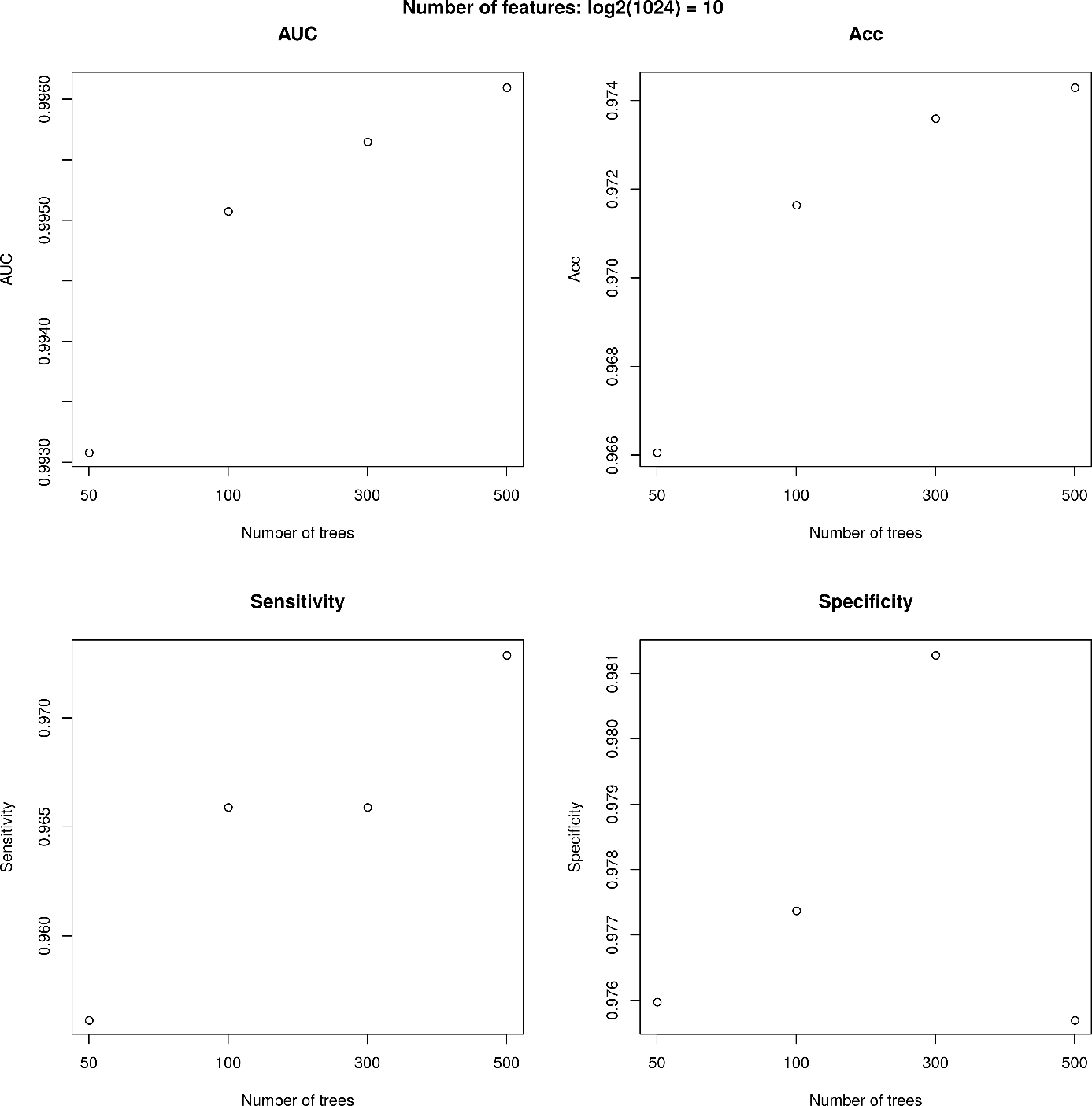


**Figure S7** The dependence of T_CP_ AUC, Acc, SN and SP on the number of trees in the RF (n_estimators keyword in sklearn.ensemble.RandomForestClassifier) for the number of features considered when looking for the best split (max_features keyword) equaling to log_2_(1024), i.e. for 10 features.


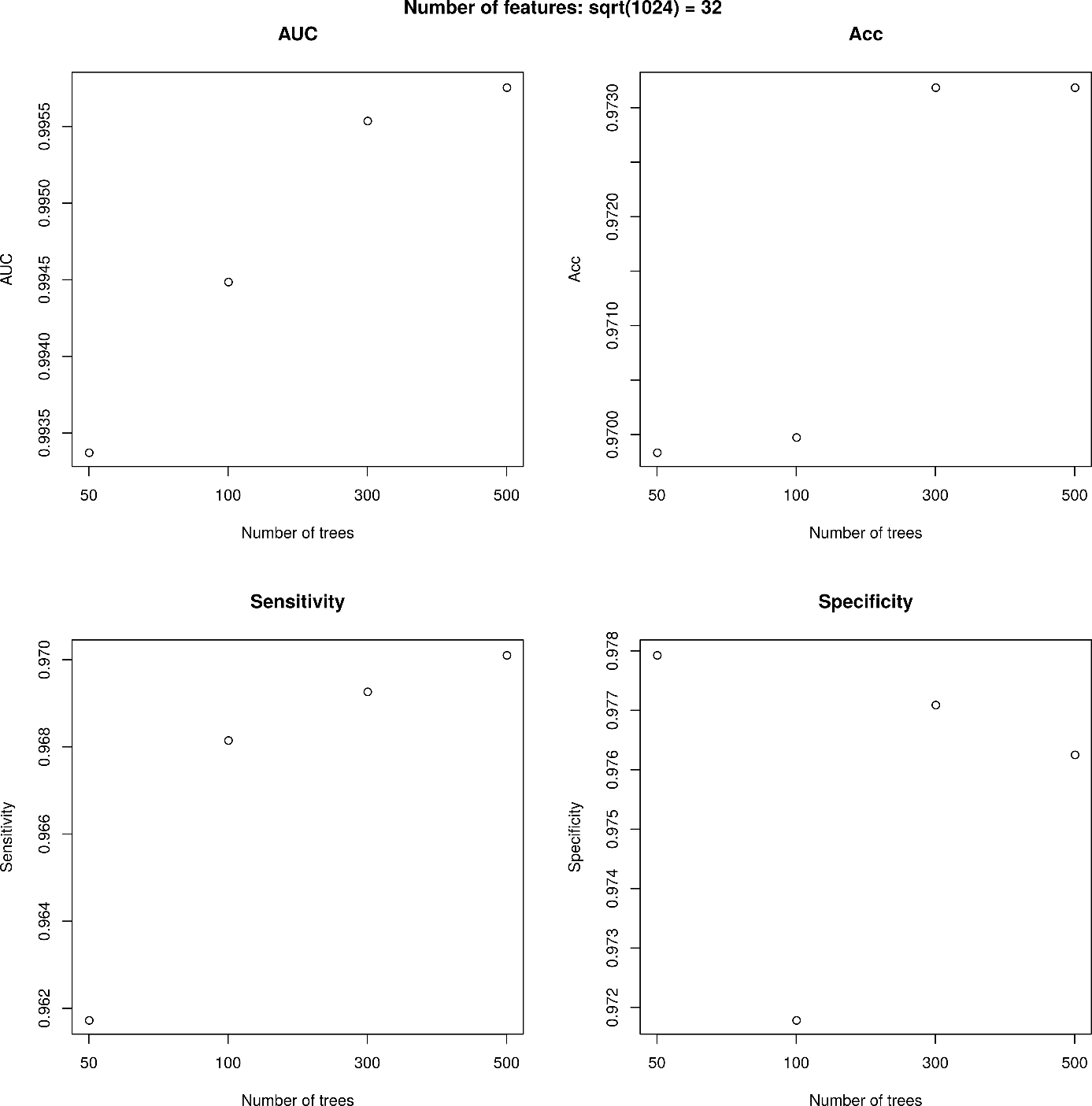


**Figure S8** The dependence of T_CP_ AUC, Acc, SN and SP on the number of trees in the RF (n_estimators keyword in sklearn.ensemble.RandomForestClassifier) for the number of features considered when looking for the best split (max_features keyword) equaling to sqrt(1024), i.e. for 32 features.


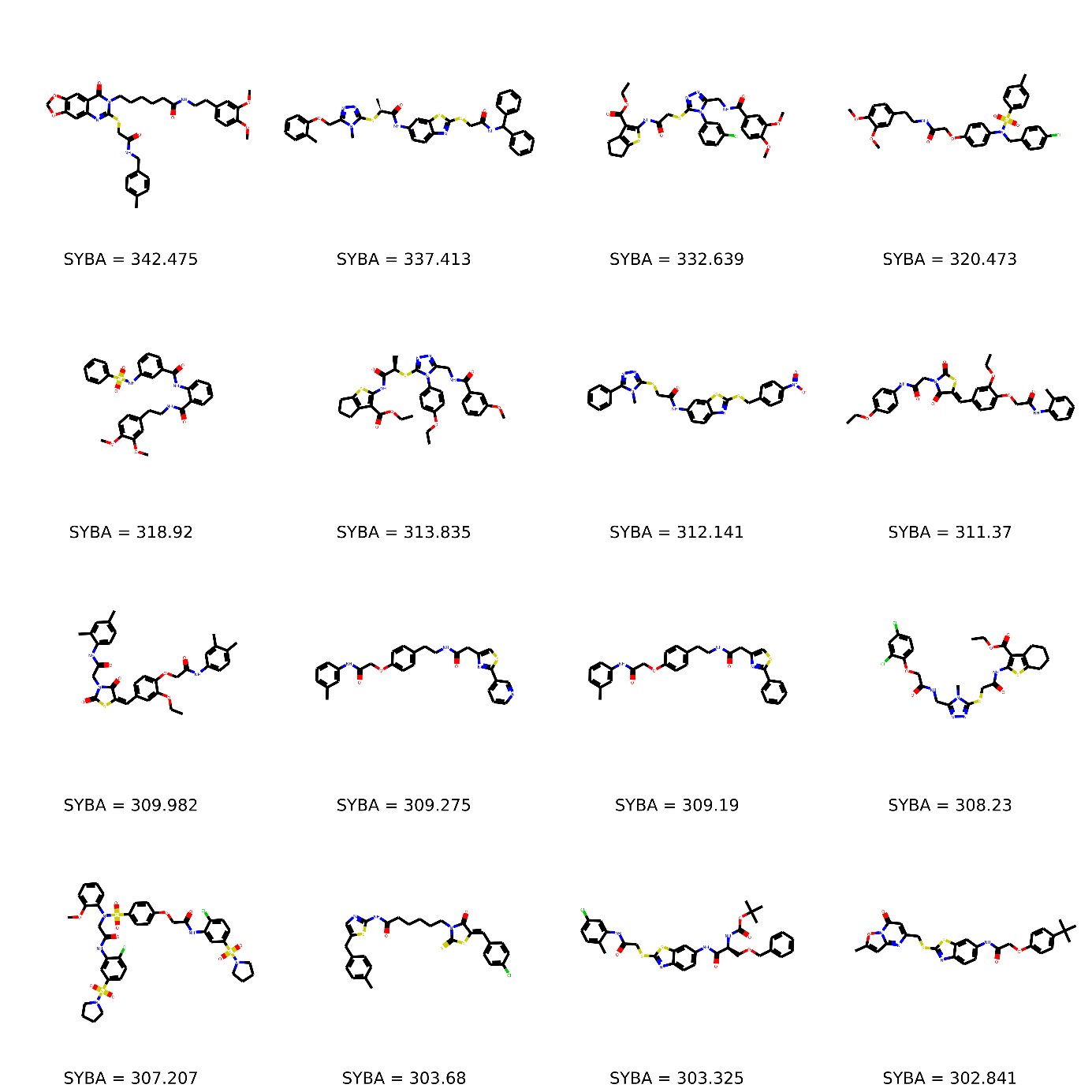


**Figure S9** 16 training set ES compounds with the highest SYBA score.


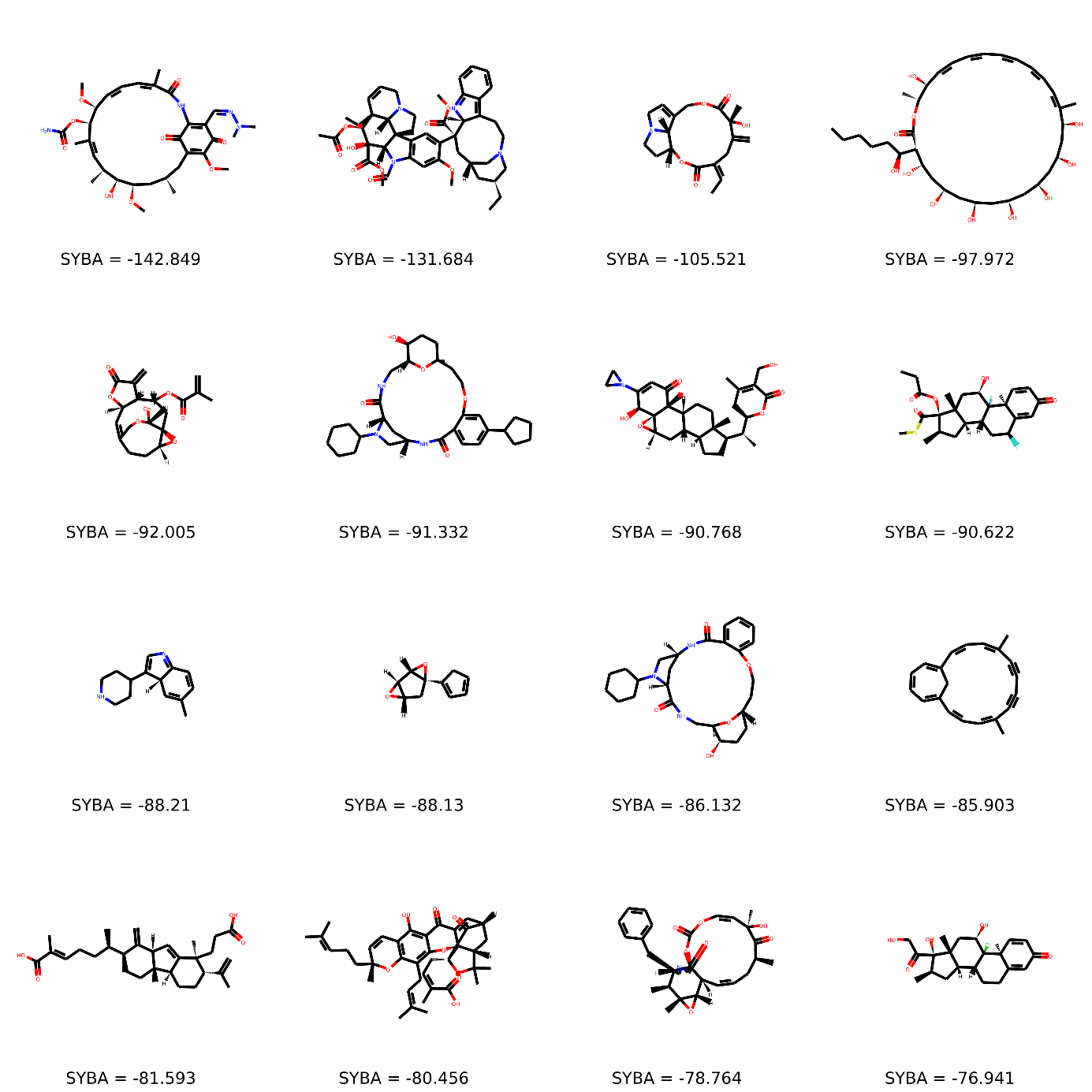


**Figure S10** 16 training set ES compounds with the the lowest SYBA score. These compounds represent mispredictions by the SYBA model.


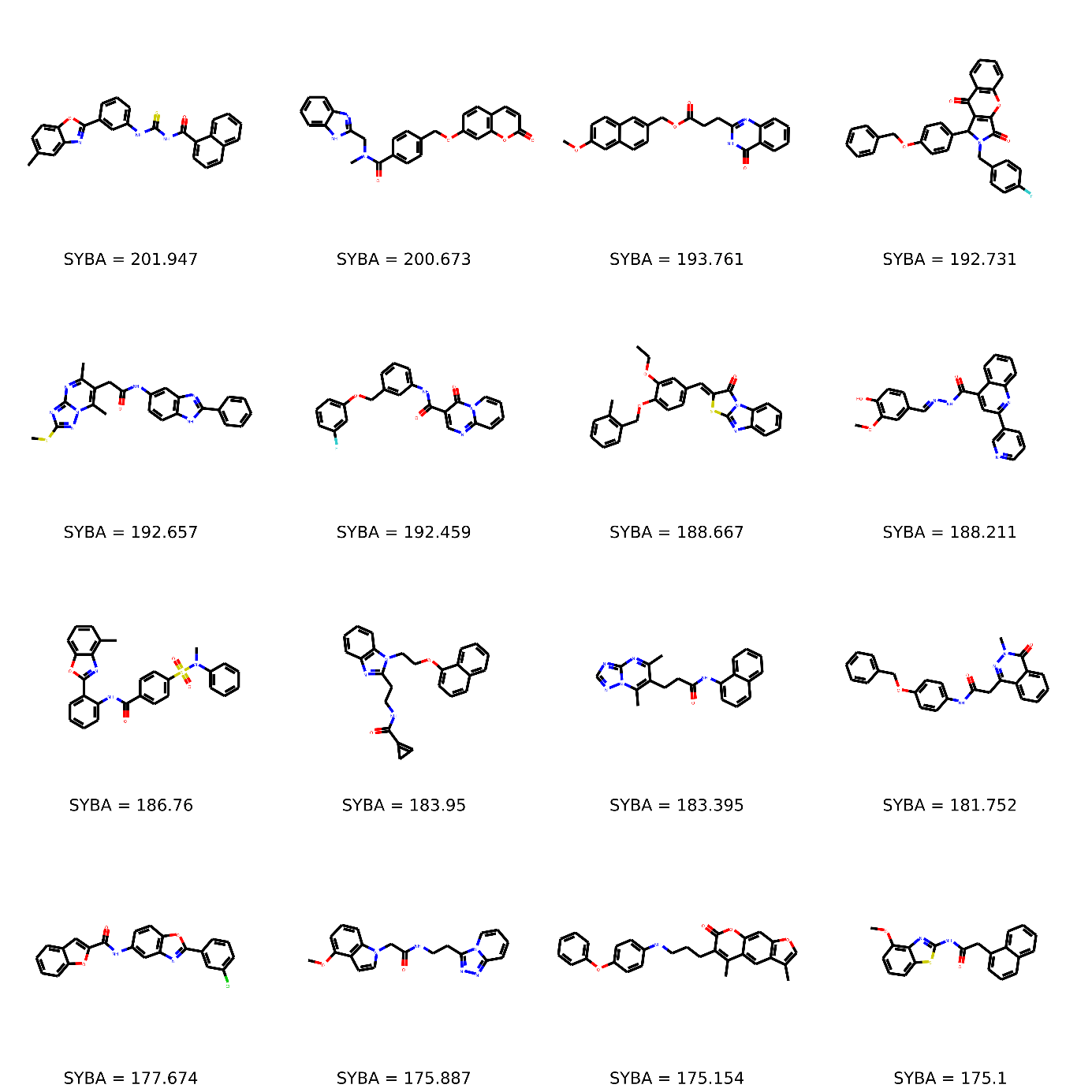


**Figure S11** 16 training set HS compounds with the highest SYBA score. These compounds represent mispredictions by the SYBA model.


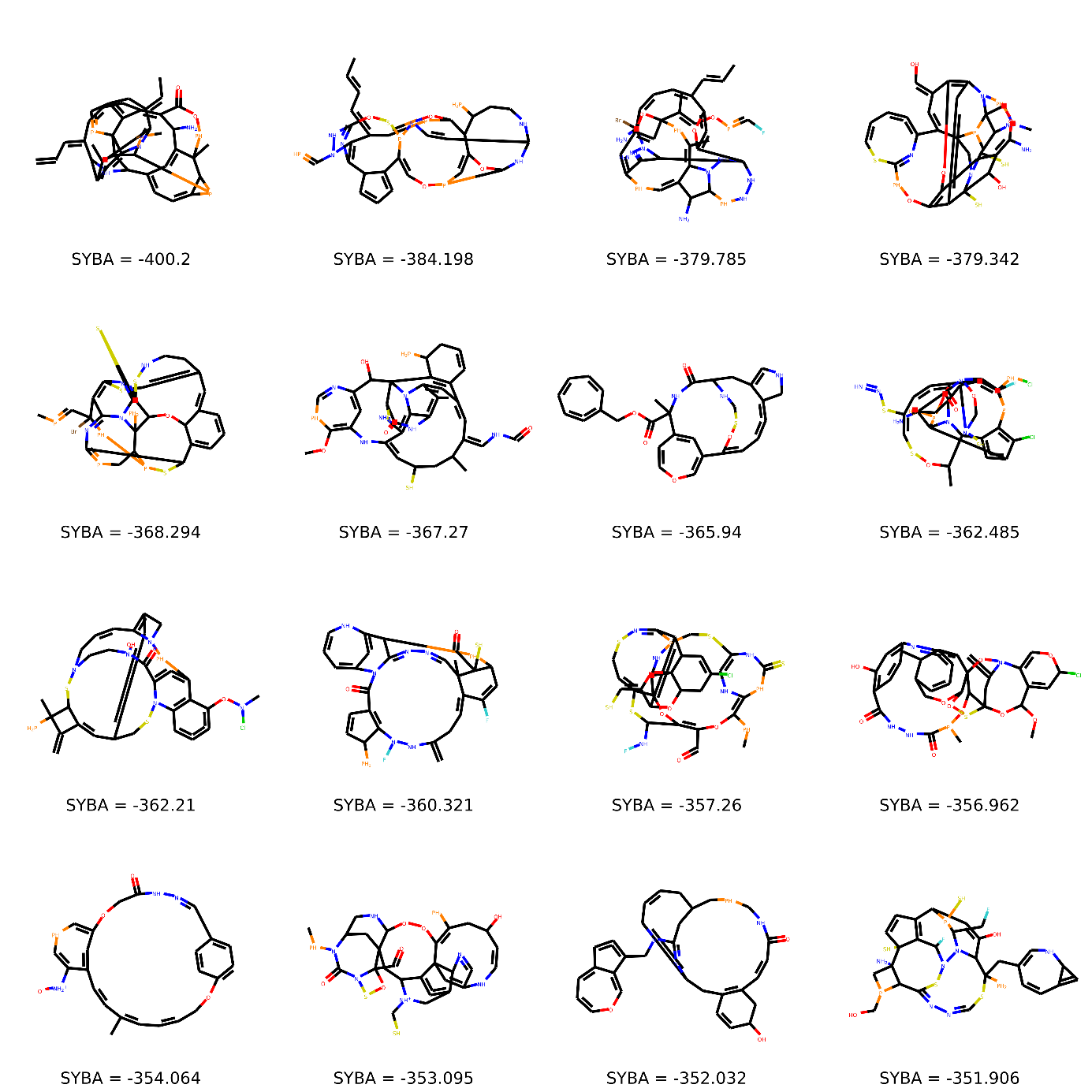


**Figure S12** 16 training set HS compounds with the lowest SYBA score.

**
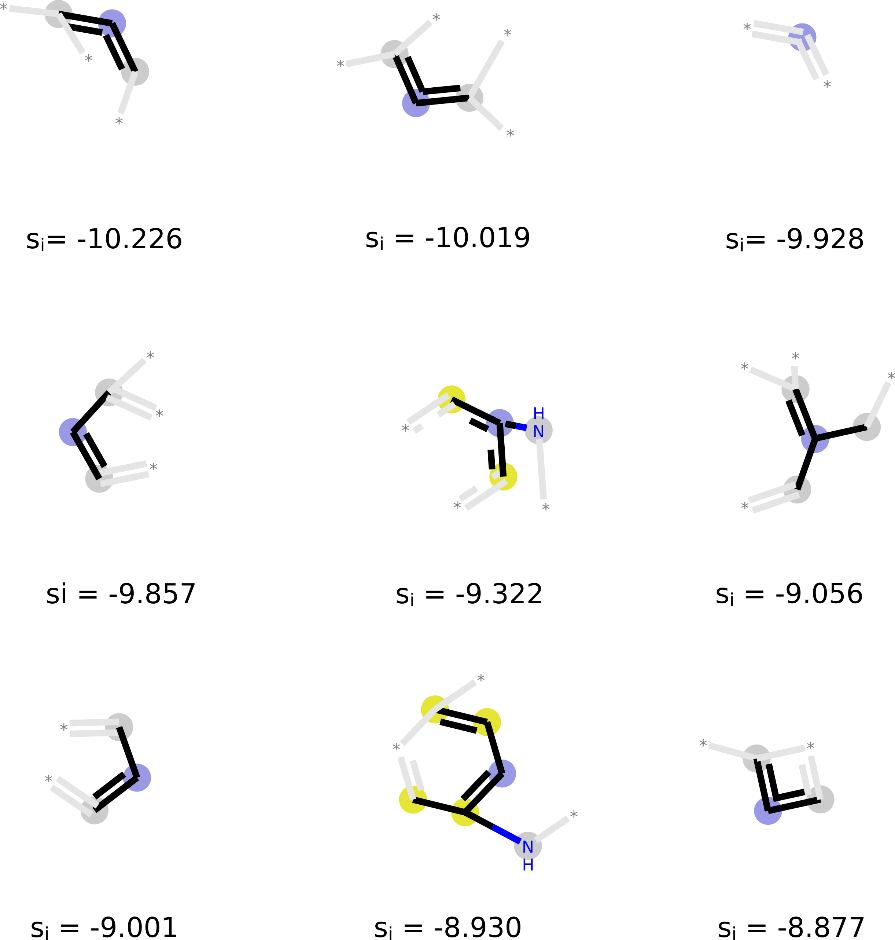
**

**Figure S13** Fragments with very low SYBA score contribution s_i_.


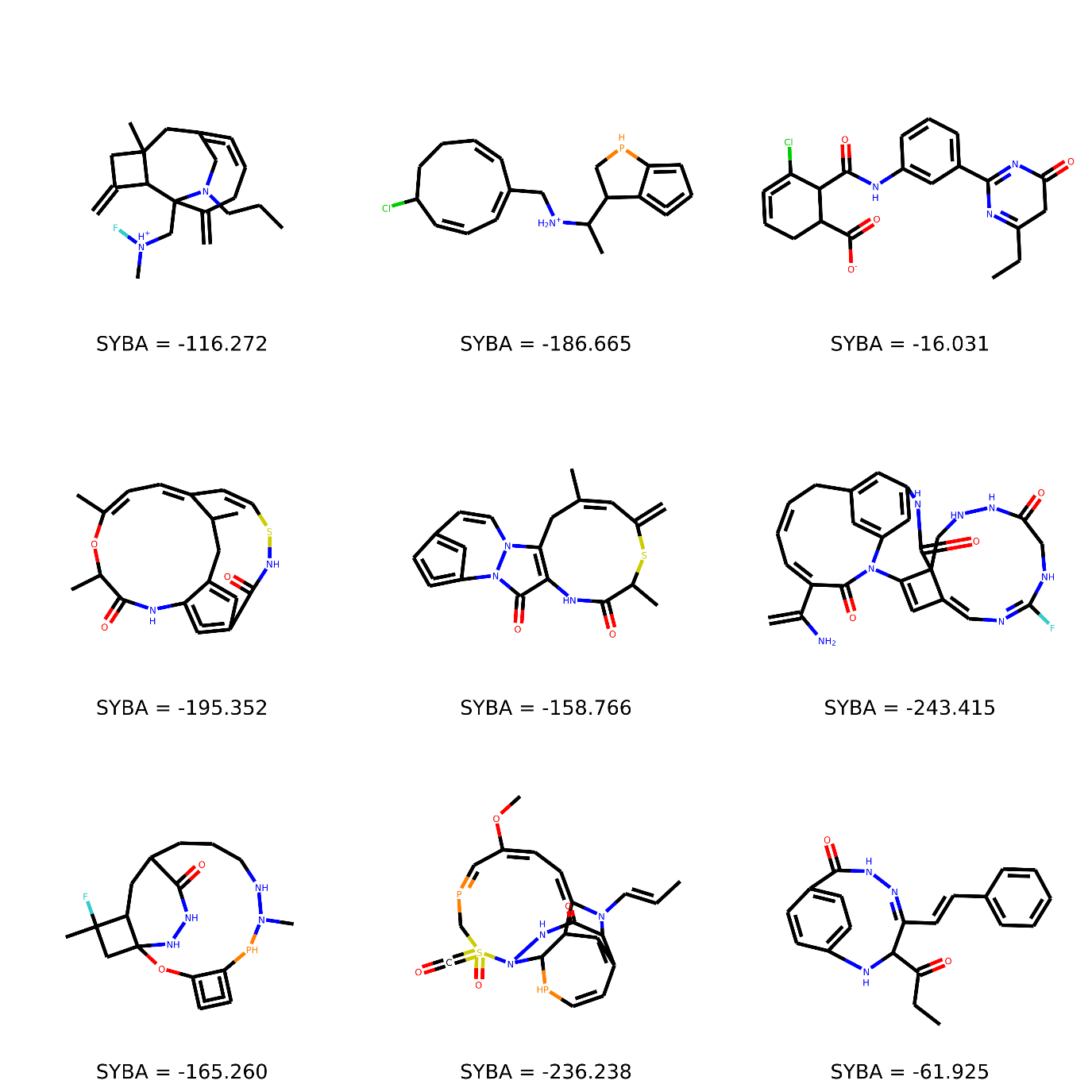


**Figure S14** Training HS compounds containing fragments with very low SYBA score contribution that are shown at Figure S13.


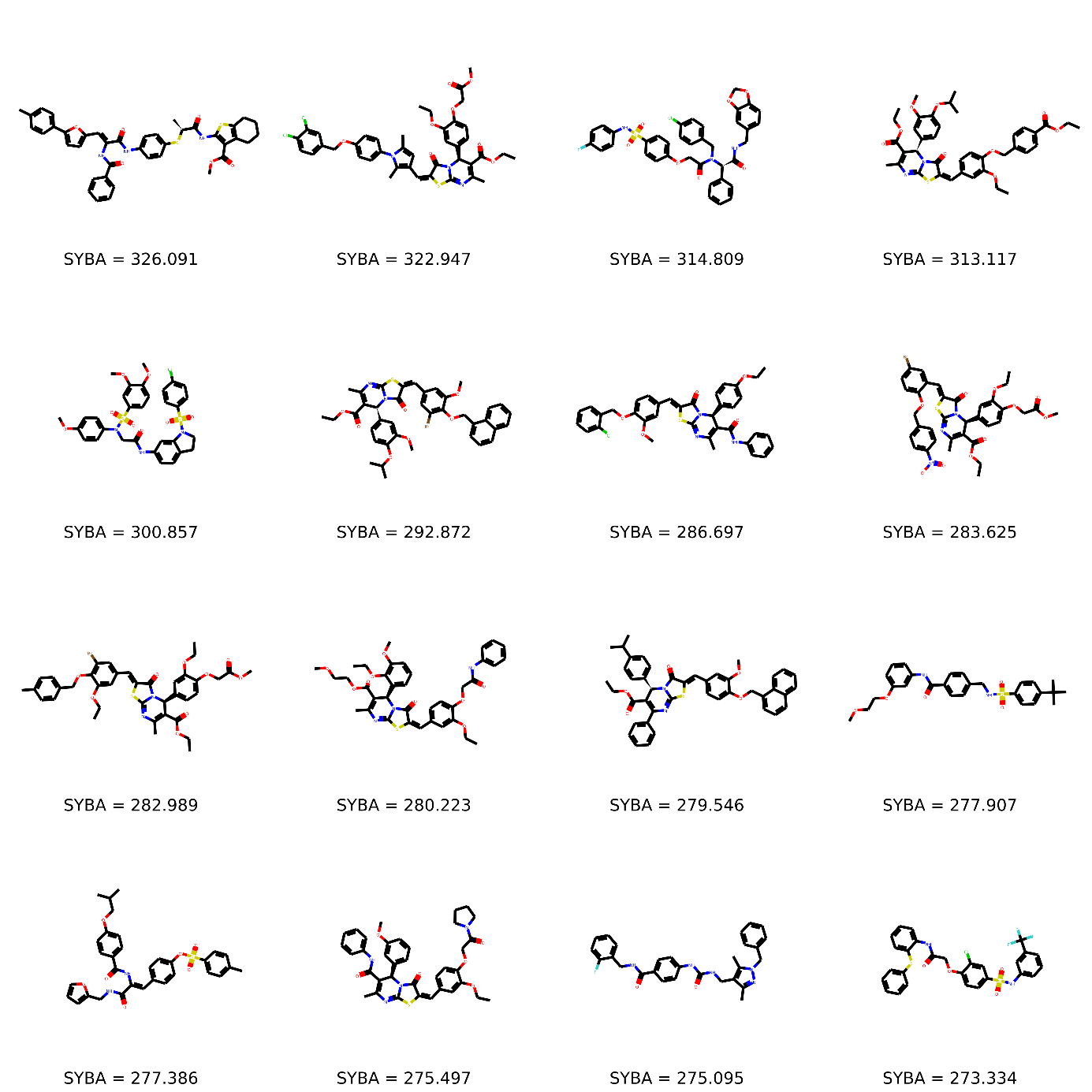


**Figure S15** 16 T_MC_ ES compounds with the highest SYBA score.


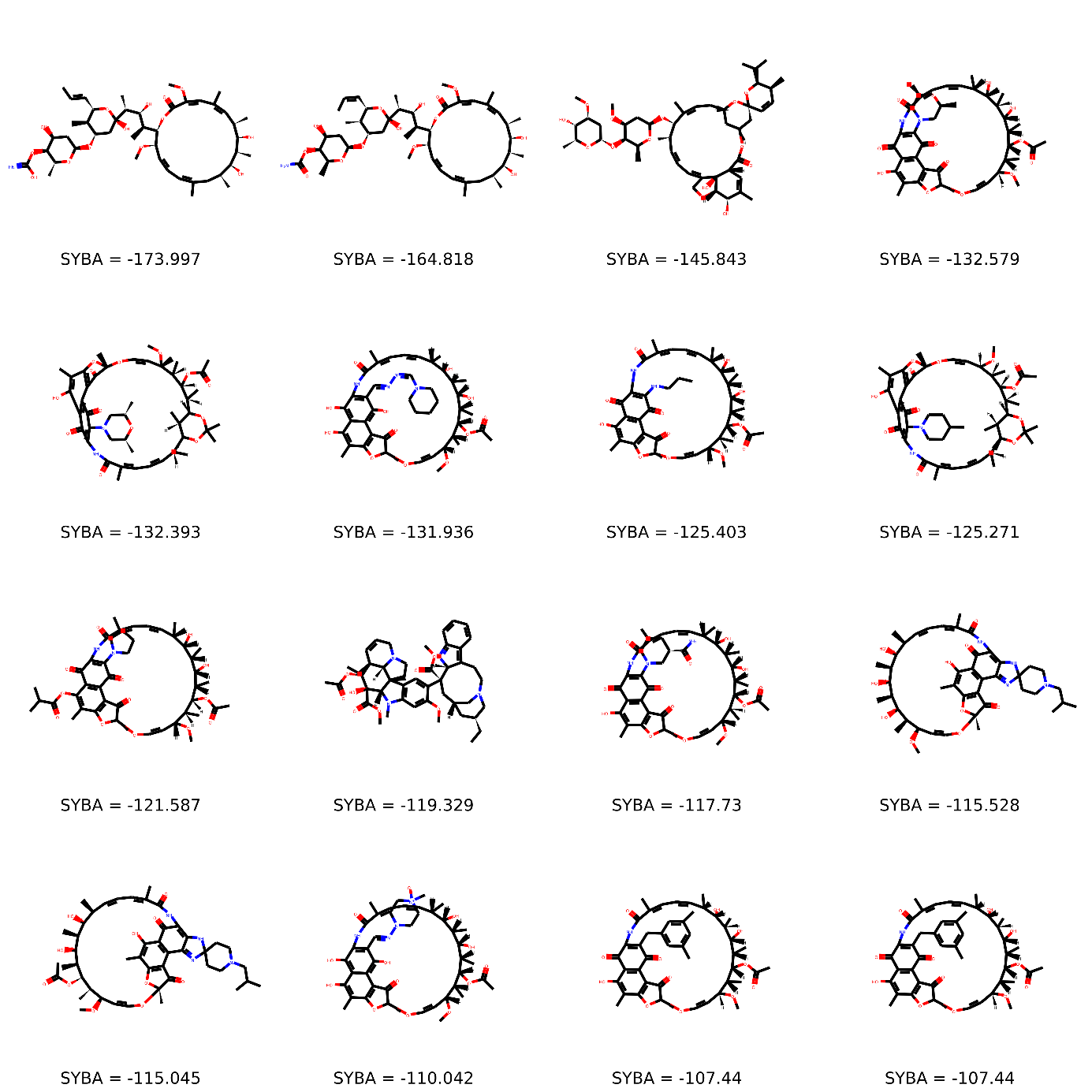


**Figure S16** 16 T_MC_ ES compounds with the lowest SYBA score. These compounds represent mispredictions by the SYBA model.


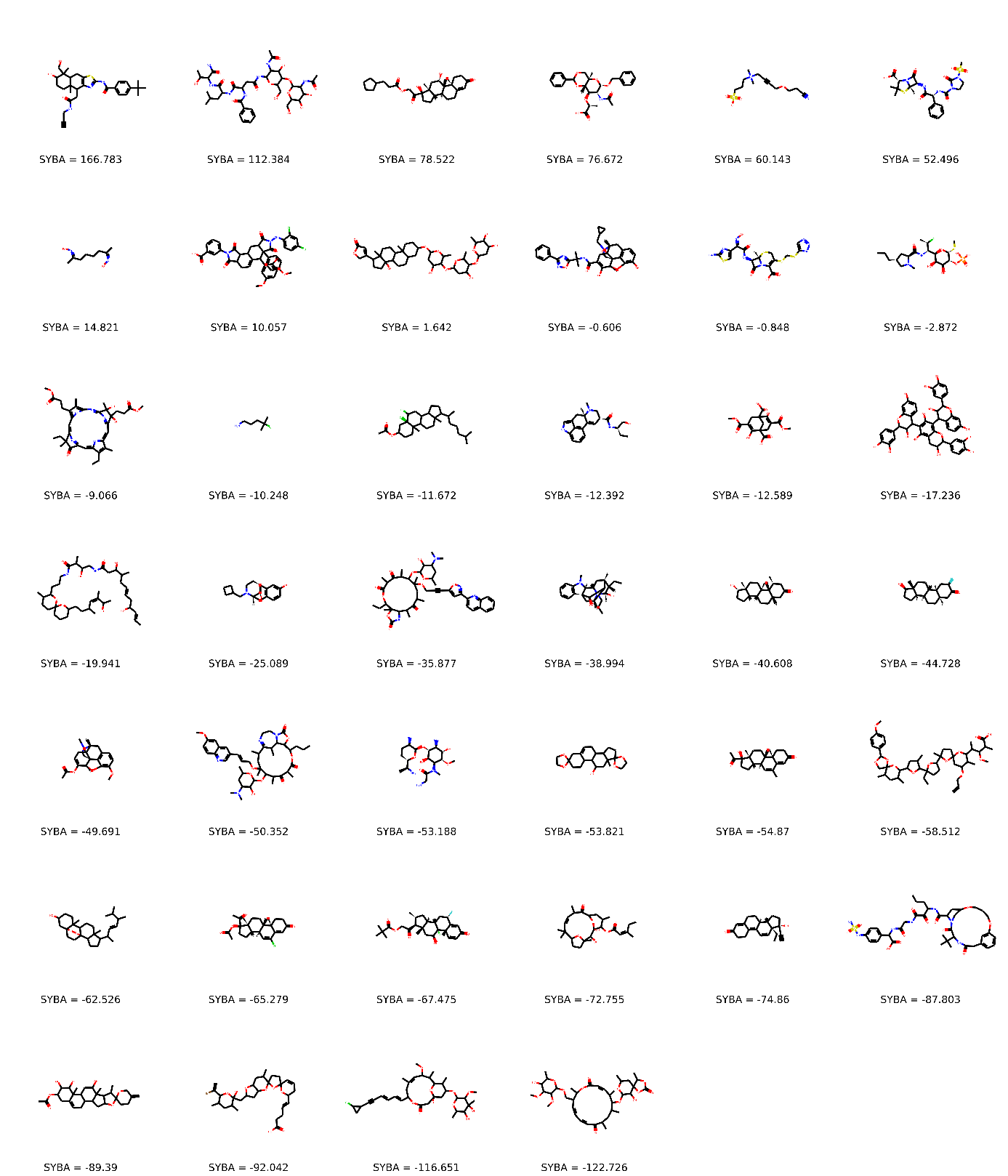


**Figure S17** All (40) T_MC_ HS compounds. Compounds with positive SYBA score represent mispredictions by the SYBA model.


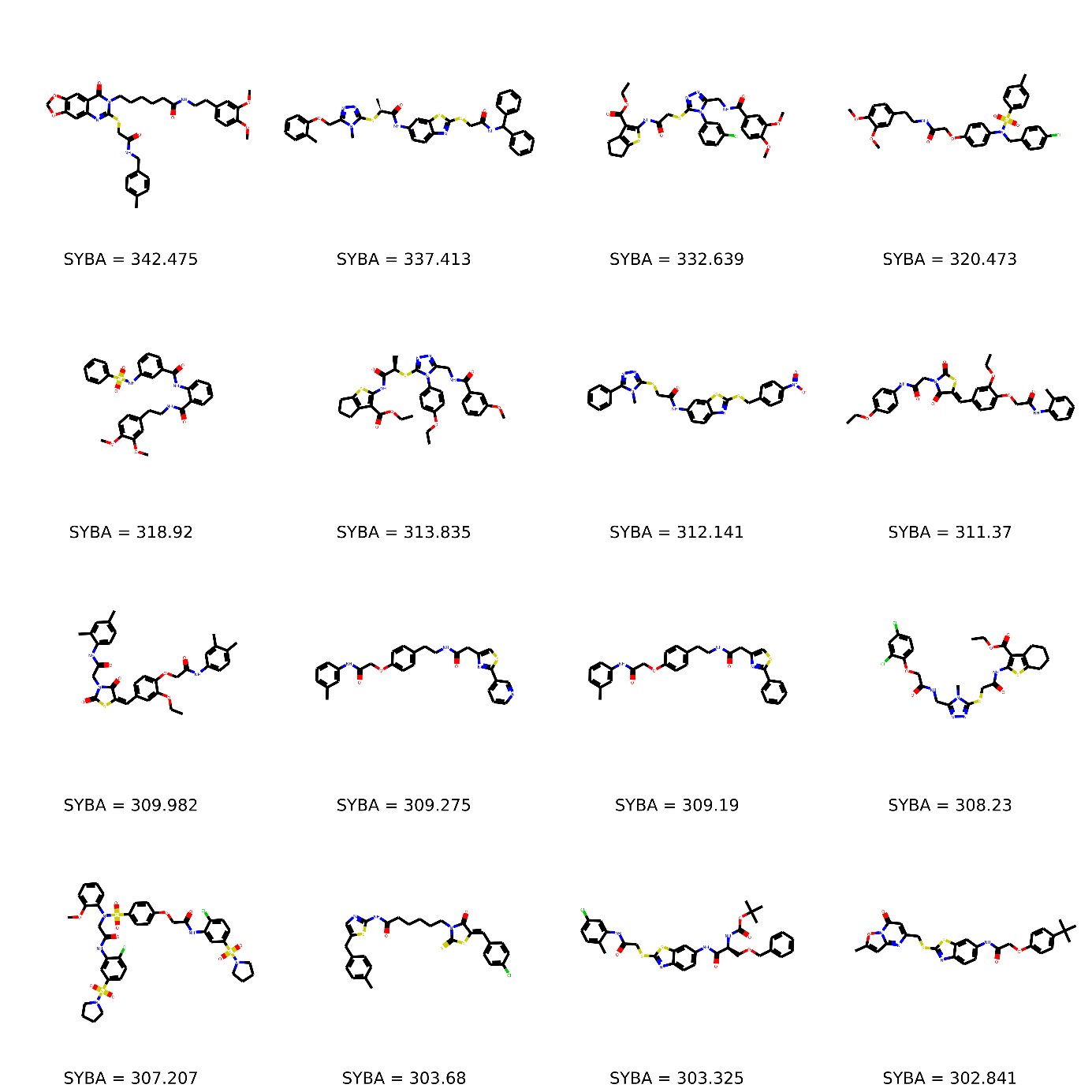


**Figure S18** 16 T_CP_ ES compounds with the highest SYBA score.


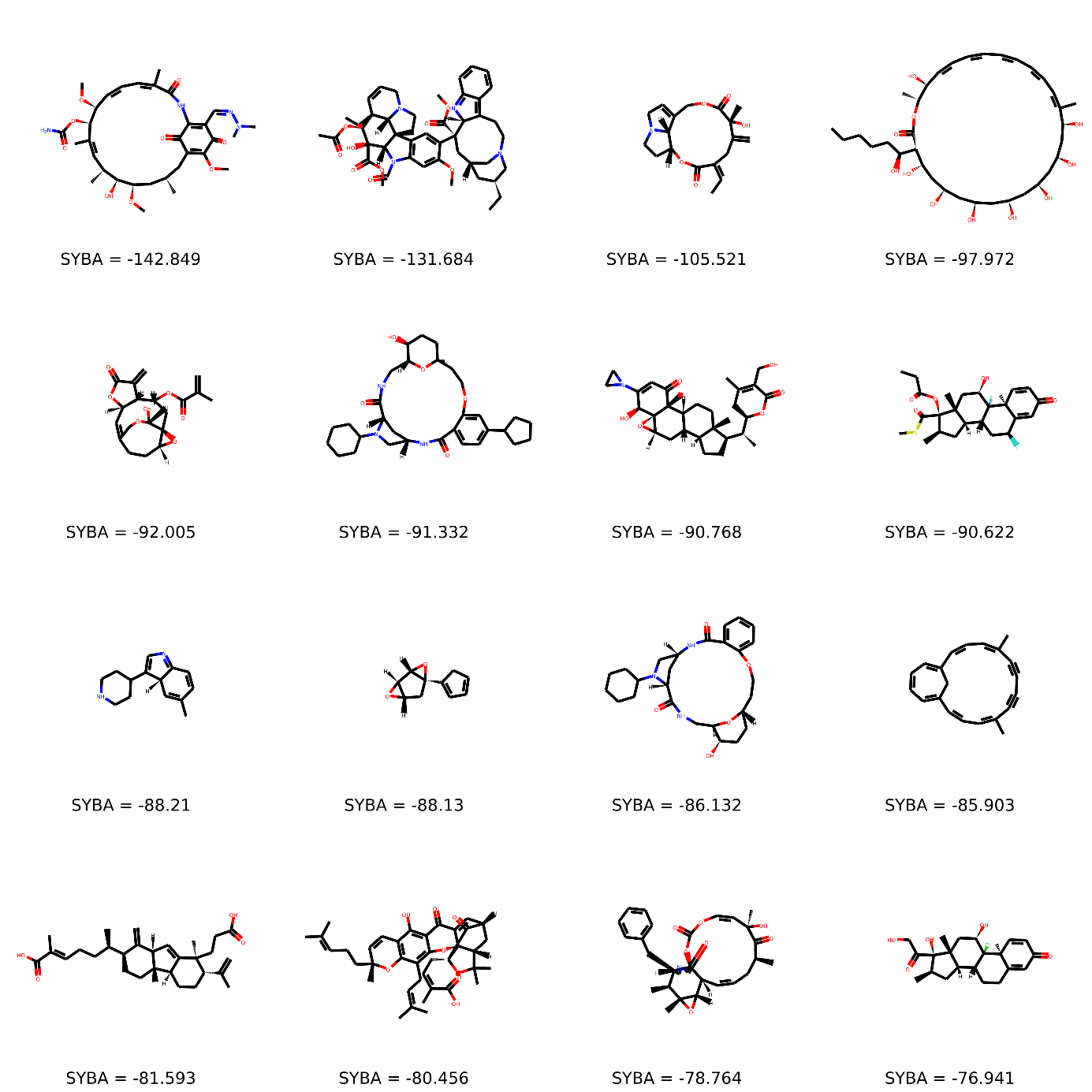


**Figure S19** 16 T_CP_ ES compounds with the lowest SYBA score. These compounds represent mispredictions by the SYBA model.


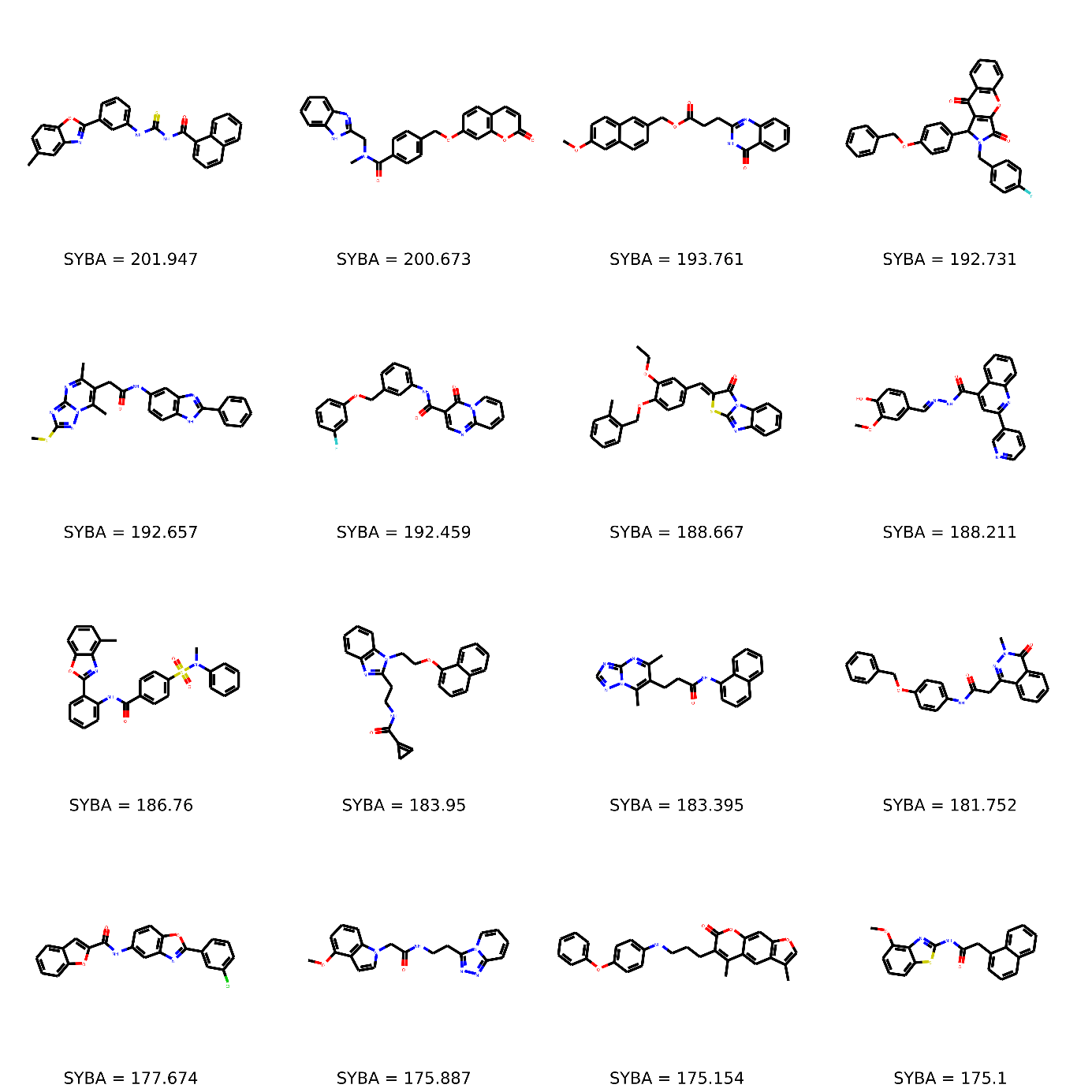


**Figure S20** 16 T_CP_ HS compounds with the highest SYBA score. These compounds represent mispredictions by the SYBA model.


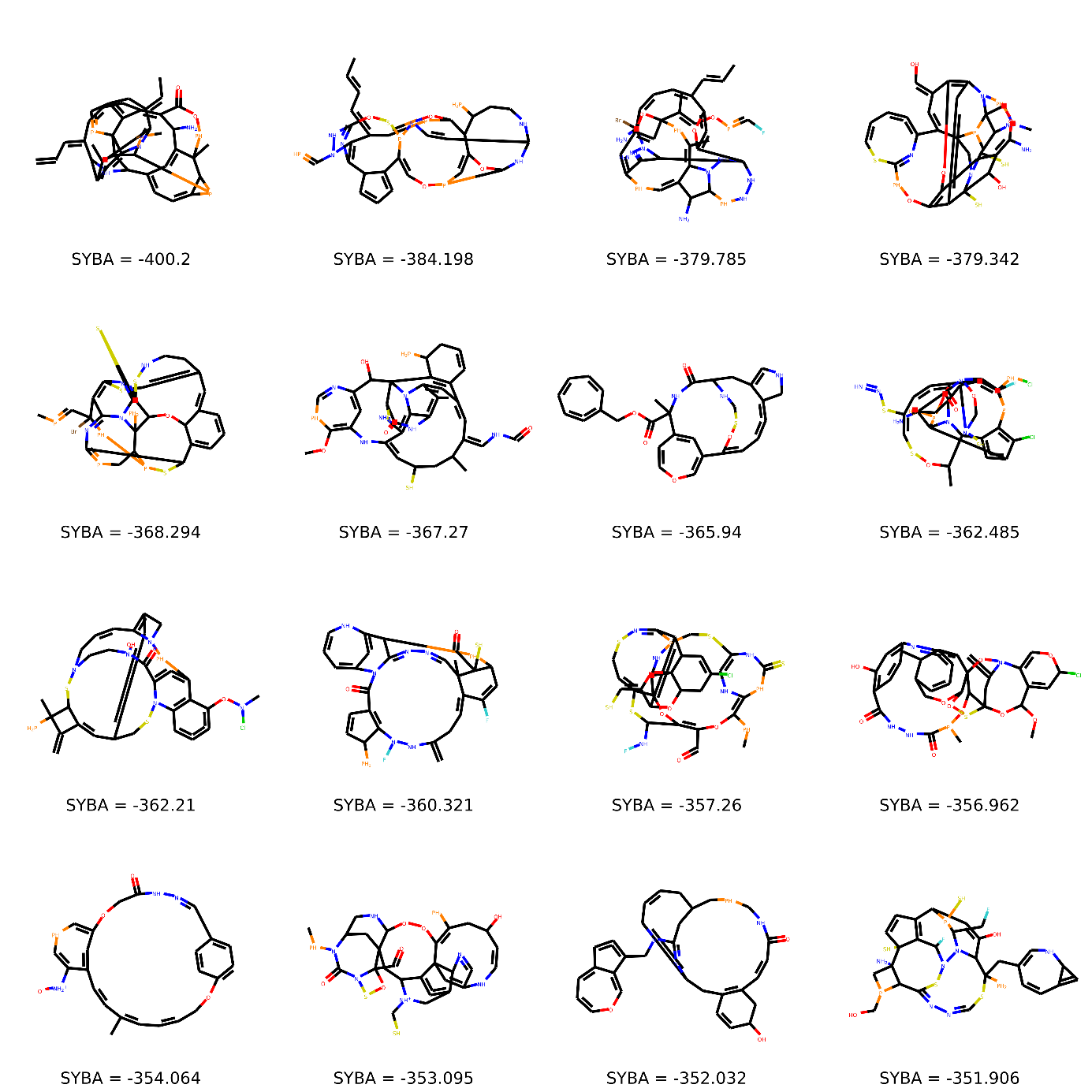


**Figure S21** 16 T_CP_ HS compounds with the lowest SYBA score.

| \|  \| **Actual ES** \| **Actual HS** \| \| --- \| --- \| --- \| \| **Predicted ES** \| 33 \| 9 \| \| **Predicted HS** \| 7 \| 31 \|   SYBA, default threshold (0.0)   \|  \| **Actual ES** \| **Actual HS** \| \| --- \| --- \| --- \| \| **Predicted ES** \| 35 \| 28 \| \| **Predicted HS** \| 5 \| 12 \|   SAScore, default threshold (6.0)   \|  \| **Actual ES** \| **Actual HS** \| \| --- \| --- \| --- \| \| **Predicted ES** \| 31 \| 9 \| \| **Predicted HS** \| 9 \| 31 \|   RF, default threshold (0.5) | \|  \| **Actual ES** \| **Actual HS** \| \| --- \| --- \| --- \| \| **Predicted ES** \| 40 \| 6 \| \| **Predicted HS** \| 0 \| 34 \|   SYBA, optimal threshold (20.1)     \|  \| **Actual ES** \| **Actual HS** \| \| --- \| --- \| --- \| \| **Predicted ES** \| 31 \| 2 \| \| **Predicted HS** \| 9 \| 38 \|   SAScore, optimal threshold (3.7)   \|  \| **Actual ES** \| **Actual HS** \| \| --- \| --- \| --- \| \| **Predicted ES** \| 31 \| 8 \| \| **Predicted HS** \| 9 \| 32 \|   RF, optimal threshold (0.5)   \|  \| **Actual ES** \| **Actual HS** \| \| --- \| --- \| --- \| \| **Predicted ES** \| 25 \| 16 \| \| **Predicted HS** \| 15 \| 24 \|   SCScore, optimal threshold (4.0) |
| --- | --- | --- | --- | --- | --- | --- | --- | --- | --- | --- | --- | --- | --- | --- | --- | --- | --- | --- | --- | --- | --- | --- | --- | --- | --- | --- | --- | --- | --- | --- | --- | --- | --- | --- | --- | --- | --- | --- | --- | --- | --- | --- | --- | --- | --- | --- | --- | --- | --- | --- | --- | --- | --- | --- | --- | --- | --- | --- | --- | --- | --- | --- | --- | --- |

Panel S1 Confusion matrices of the classification of the manually curated T_MC_ test set. Out of possible 30 T_MC_ sets differing in T_MC+_ compounds, confusion matrices are reported for the T_MC_ set with the smallest SYBA *AUC* (0.830).

| \|  \| **Actual ES** \| **Actual HS** \| \| --- \| --- \| --- \| \| **Predicted ES** \| 40 \| 9 \| \| **Predicted HS** \| 0 \| 31 \|   SYBA, default threshold (0.0)   \|  \| **Actual ES** \| **Actual HS** \| \| --- \| --- \| --- \| \| **Predicted ES** \| 40 \| 28 \| \| **Predicted HS** \| 0 \| 12 \|   SAScore, default threshold (6.0)   \|  \| **Actual ES** \| **Actual HS** \| \| --- \| --- \| --- \| \| **Predicted ES** \| 38 \| 9 \| \| **Predicted HS** \| 2 \| 31 \|   RF, default threshold (0.5) | \|  \| **Actual ES** \| **Actual HS** \| \| --- \| --- \| --- \| \| **Predicted ES** \| 40 \| 6 \| \| **Predicted HS** \| 0 \| 34 \|   SYBA, optimal threshold (20.1)     \|  \| **Actual ES** \| **Actual HS** \| \| --- \| --- \| --- \| \| **Predicted ES** \| 36 \| 6 \| \| **Predicted HS** \| 4 \| 34 \|   SAScore, optimal threshold (4.2)   \|  \| **Actual ES** \| **Actual HS** \| \| --- \| --- \| --- \| \| **Predicted ES** \| 37 \| 8 \| \| **Predicted HS** \| 3 \| 32 \|   RF, optimal threshold (0.5)   \|  \| **Actual ES** \| **Actual HS** \| \| --- \| --- \| --- \| \| **Predicted ES** \| 32 \| 21 \| \| **Predicted HS** \| 8 \| 19 \|   SCScore, optimal threshold (3.6) |
| --- | --- | --- | --- | --- | --- | --- | --- | --- | --- | --- | --- | --- | --- | --- | --- | --- | --- | --- | --- | --- | --- | --- | --- | --- | --- | --- | --- | --- | --- | --- | --- | --- | --- | --- | --- | --- | --- | --- | --- | --- | --- | --- | --- | --- | --- | --- | --- | --- | --- | --- | --- | --- | --- | --- | --- | --- | --- | --- | --- | --- | --- | --- | --- | --- |

Panel S2 Confusion matrices of the classification of the manually curated T_MC_ test set. Out of possible 30 T_MC_ sets differing in T_MC+_ compounds, confusion matrices are reported for the TMC set with the the highest SYBA *AUC* (0.943).

| \|  \| **Actual ES** \| **Actual HS** \| \| --- \| --- \| --- \| \| **Predicted ES** \| 3312 \| 0 \| \| **Predicted HS** \| 269 \| 3581 \|   SYBA, default threshold (0.0)   \|  \| **Actual ES** \| **Actual HS** \| \| --- \| --- \| --- \| \| **Predicted ES** \| 3579 \| 2395 \| \| **Predicted HS** \| 2 \| 1186 \|   SAScore, default threshold (6.0)   \|  \| **Actual ES** \| **Actual HS** \| \| --- \| --- \| --- \| \| **Predicted ES** \| 2806 \| 1 \| \| **Predicted HS** \| 775 \| 3580 \|   RF, default threshold (0.5) | \|  \| **Actual ES** \| **Actual HS** \| \| --- \| --- \| --- \| \| **Predicted ES** \| 3528 \| 31 \| \| **Predicted HS** \| 53 \| 3550 \|   SYBA, optimal threshold (-18.6)     \|  \| **Actual ES** \| **Actual HS** \| \| --- \| --- \| --- \| \| **Predicted ES** \| 3532 \| 26 \| \| **Predicted HS** \| 49 \| 3555 \|   SAScore, optimal threshold (4.5)   \|  \| **Actual ES** \| **Actual HS** \| \| --- \| --- \| --- \| \| **Predicted ES** \| 3439 \| 50 \| \| **Predicted HS** \| 142 \| 3531 \|   RF, optimal threshold (0.2)   \|  \| **Actual ES** \| **Actual HS** \| \| --- \| --- \| --- \| \| **Predicted ES** \| 1787 \| 986 \| \| **Predicted HS** \| 1794 \| 2595 \|   SCScore, optimal threshold (3.1) |
| --- | --- | --- | --- | --- | --- | --- | --- | --- | --- | --- | --- | --- | --- | --- | --- | --- | --- | --- | --- | --- | --- | --- | --- | --- | --- | --- | --- | --- | --- | --- | --- | --- | --- | --- | --- | --- | --- | --- | --- | --- | --- | --- | --- | --- | --- | --- | --- | --- | --- | --- | --- | --- | --- | --- | --- | --- | --- | --- | --- | --- | --- | --- | --- | --- |

Panel S3 Confusion matrices of the classification of the computationally picked T_CP_ test set.

| \|  \| **Actual ES** \| **Actual HS** \| \| --- \| --- \| --- \| \| **Predicted ES** \| 692 058 \| 321 483 \| \| **Predicted HS** \| 1 295 \| 371 870 \|   SAScore, default threshold (6.0) | \|  \| **Actual ES** \| **Actual HS** \| \| --- \| --- \| --- \| \| **Predicted ES** \| 648 301 \| 47 391 \| \| **Predicted HS** \| 45 052 \| 645 962 \|   SAScore, optimal threshold (4.4)     \|  \| **Actual ES** \| **Actual HS** \| \| --- \| --- \| --- \| \| **Predicted ES** \| 391 138 \| 220 575 \| \| **Predicted HS** \| 302 215 \| 472 778 \|   SCScore, optimal threshold (3.7) |
| --- | --- | --- | --- | --- | --- | --- | --- | --- | --- | --- | --- | --- | --- | --- | --- | --- | --- | --- | --- | --- | --- | --- | --- | --- | --- | --- | --- | --- |

Panel S4 Confusion matrices of the classification of the S training set.
